# *shox2* is required for vestibular statoacoustic neuron development

**DOI:** 10.1242/bio.059599

**Published:** 2023-01-03

**Authors:** Alejandra S. Laureano, Kathleen Flaherty, Anna-Maria Hinman, Azadeh Jadali, Tetsuya Nakamura, Shin-ichi Higashijima, Hatim E. Sabaawy, Kelvin Y. Kwan

**Affiliations:** ^1^ Department of Cell Biology & Neuroscience, Rutgers University, Piscataway, NJ 08854, USA; ^2^ Stem Cell Research Center and Keck Center for Collaborative Neuroscience, Rutgers University, NJ 08854, USA; ^3^ Department of Comparative Medicine Resources, Rutgers University, Piscataway, NJ 08854, USA; ^4^ Department of Genetics, Rutgers University, Piscataway, NJ 08854, USA; ^5^ Institutes of Natural Sciences, Exploratory Research Center on Life and Living Systems, Okazaki, Aichi 444-8787, Japan; ^6^ Department of Medicine, Division of Medical Oncology, University of Colorado Anschutz Medical Campus, Aurora, CO 80045, USA; ^7^ Department of Medicine RBHS-Robert Wood Johnson Medical School, Rutgers University, Piscataway, NJ 08854, USA

**Keywords:** Inner ear, Vestibular neuron, Statoacoustic ganglion neuron

## Abstract

Homeobox genes act at the top of genetic hierarchies to regulate cell specification and differentiation during embryonic development. We identified the short stature homeobox domain 2 (*shox2*) transcription factor that is required for vestibular neuron development. *shox2* transcripts are initially localized to the otic placode of the developing inner ear where neurosensory progenitors reside. To study *shox2* function, we generated CRISPR-mediated mutant *shox2* fish. Mutant embryos display behaviors associated with vestibular deficits and showed reduced number of anterior statoacoustic ganglion neurons that innervate the utricle, the vestibular organ in zebrafish. Moreover, a *shox2*-reporter fish showed labeling of developing statoacoustic ganglion neurons in the anterior macula of the otic vesicle. Single cell RNA-sequencing of cells from the developing otic vesicle of *shox2* mutants revealed altered otic progenitor profiles, while single molecule *in situ* assays showed deregulated levels of transcripts in developing neurons. This study implicates a role for *shox2* in development of vestibular but not auditory statoacoustic ganglion neurons.

## Teaser

**Summary:** Mutant shox2 fish display behaviors associated with vestibular deficits, show reduced number of anterior statoacoustic ganglion neurons that innervate the utricle and have altered otic cell populations defined by single cell RNA-seq.

## INTRODUCTION

Homeobox genes are master regulators in genetic hierarchies and regulate aspects of cell fate and differentiation in animals. These proteins are sequence-specific DNA binding proteins that control gene expression and display complex expression patterns in embryonic tissues. Combinatorial interactions among transcription factors forms genomic regulatory networks during development and are critical for tissue differentiation (Davidson et al., 2002). We identified the zebrafish *short stature homeobox domain 2* (*shox2*) gene as an early expressing transcription factor in the developing inner ear. The human SHOX (short stature homeobox) gene family consists of two members, SHOX and SHOX2. Intriguingly, a SHOX ortholog does not exist in the murine genome. Mice have lost *Shox* along with other pseudoautosomal genes but retain the *Shox2* paralog (Gianfrancesco et al., 2001). In zebrafish, both *shox* and *shox2* are present. These two zebrafish genes exhibit overlapping and distinct expression patterns in many developing organs. Using *in situ* hybridization, *shox2* is detected in the otic placode, diencephalon, cranial ganglion neurons, hindbrain, and optic tectum (Thisse and Thisse, 2004). While *shox2* expression has been identified in the zebrafish inner ear, its functional role in the inner ear has not been described.

The zebrafish inner ear develops from the otic placode, a thickened epithelial structure adjacent to rhombomeres 5 and 6 in the caudal region of the hindbrain. The otic placode then cavitates to form the otic vesicle. Secreted factors from hindbrain and mesenchyme provide inductive signals to mediate inner ear development (Haddon and Lewis, 1996). Fibroblast growth factor (Fgf), and Hedgehog signaling provide cues to pattern the otic vesicle and establish the neurosensory domain (Hammond et al., 2003; Hammond and Whitfield, 2011; Hans et al., 2007). Development of neurosensory progenitors rely on highly conserved network of transcription factors during statoacoustic ganglion neurons (SAG) development (Chatterjee et al., 2010); 2015; Schimmang, 2013). Subsequent diversification of progenitors into distinct inner ear cell types is a key step for inner ear function. The otic epithelium initially segregates into two independent domains, a neurosensory competent domain and a non-neural domain (Abello and Alsina, 2007; Gálvez et al., 2017). Neurosensory progenitors become either neuronal or sensory precursors. Once neuronal progenitors have delaminated, the remaining progenitors in the otic epithelium develop into either sensory hair cells or supporting cells. The transcription factors *foxi1* and *dlx3b/4b* play a role in acquiring neuronal or sensory competence and establishing the neurosensory competent domain starting at 12 h post-fertilization (hpf) (Hans et al., 2007; Solomon et al., 2003). Embryos with homozygous deletion of *dlx3b*/*4b*, and *sox9a* lose all otic sensory lineages but maintain otic neuroblast markers in remaining otic cells (Hans et al., 2007; Liu et al., 2003). Additional knockdown of *sox9b* shows a complete loss of residual otic cells and implicates *sox9b* in otic neurogenesis (Liu et al., 2003). After establishment of competence, neurogenesis begins ∼15 hpf with the specification of neural precursors (Radosevic et al., 2014). Development proceeds as neuroblasts are specified and migrate starting at 22 hpf (Haddon and Lewis, 1996; Whitfield et al., 2002). Delaminated neuroblasts differentiate into post-mitotic statoacoustic (VIIIth) ganglia (SAG) that extend peripheral projections to the sensory hair cells and central nervous system targets. These events are marked by the expression of *neurogenin1* (*neurog1*) in the anteroventral quadrant of the otic vesicle where the neurogenic domain resides (Andermann et al., 2002; Ma et al., 1998; Raft and Groves, 2015). *neurog1* expression is followed by *neurod1* expression in delaminating progenitors. Delamination from the floor of the otic vesicle ends at ∼42 hpf. *neurod1* expressing neuroblasts proliferate and migrate medially with respect to the otic vesicle before exiting the cell cycle, this process has been dubbed transit-amplification. The expression of *isl1/isl2b* characterizes the maturation stage of otic neurons. The stages of transient-amplification and maturation peak at 48 and 72 hpf, respectively (Vemaraju et al., 2012).

In this study, we generated a *shox2* mutant fish, identified the cell types that express *shox2* during inner ear neurosensory development by comparing fluorescent reporters and defined the functional consequences of ablating *shox2*. Single cell RNA-seq (scRNA-seq) and single molecule fluorescence *in situ* hybridization (smFISH) showed that deleting shox2 alters the otic progenitor population and decreases the number of vestibular but not auditory SAG neurons.

## RESULTS

### shox2 is expressed in early developing zebrafish inner ear

To confirm the expression of *shox2* at early stages of otic development, *in situ* hybridization was done. *shox2* transcripts were probed in the developing zebrafish embryos at the 18-somite stage when the otic placode appears and later time points at 18, and 24 hpf. *shox2* expression has been reported to start at 16 hpf in the otic placode and is robustly detected in the diencephalon at these time points (Thisse and Thisse, 2008). In the developing inner ear, at the 18-somite stage, *shox2 in situ* hybridization signal was observed in cells that reside in the otic placode (Fig. S1A). At 18 hpf, *shox2* transcripts were detected at the antero and posteroventral area of the hollowing otic vesicle (Fig. S1C). At 24 hpf, in the developing inner ear, *shox2* transcript were present in the statoacoustic ganglion, anterior and posterior lateral line ganglia (Fig. S1C). The expression of *shox2* at early inner ear developmental time points suggest that *shox2* is present in the otic placode and could contribute to development of inner ear cell types. To determine whether *shox2* functions in development of the inner ear, a *shox2* mutant fish was generated.

### Generation of shox2 mutant fish

**Fig. 1.**
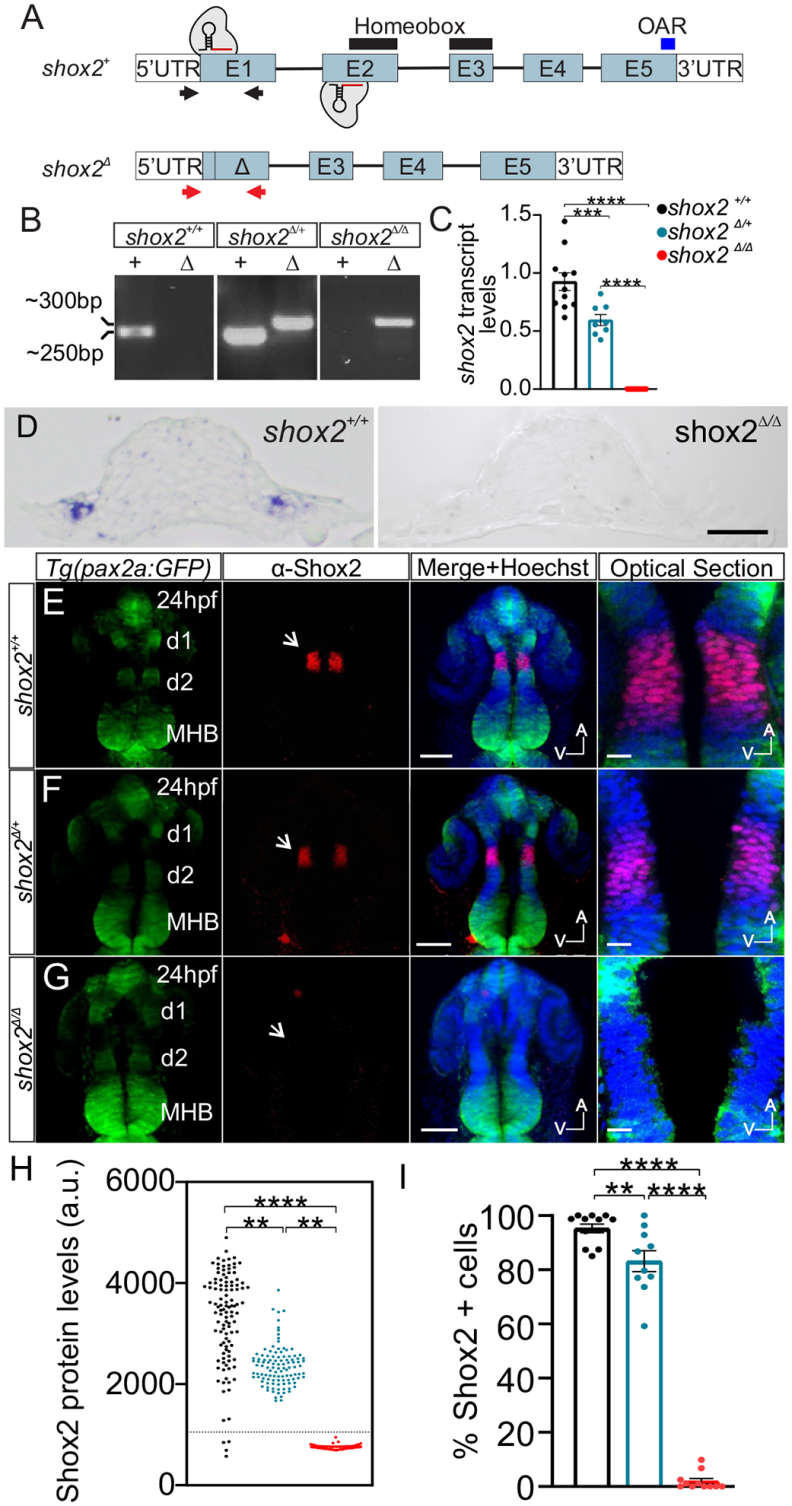
**Generating *shox2* mutant zebrafish.** (A) Genomic representation of wild-type *shox2* (*shox2^+^*) gene containing five exons (E1-5), 5′ and 3′ untranslated regions (UTRs). Black lines above exons 2 and 3 demarcate the coding region for the homeobox domain. The blue line above exon 5 marks the otp, aristaless, and rax (OAR) domain. Black arrows represent the approximate primer binding sites utilized for PCR genotyping of *shox2^+^*. The *shox2* null allele was generated using two CRISPR/Cas9 sgRNAs complexes that bind and cleave in exons 1 and 2. Repair and joining of the two cut sites deleted genomic DNA containing parts of exon1 and 2 to create the *shox2* null allele (*shox2*^Δ^). Red arrows denote the approximate binding site of primer pairs for PCR genotyping of *shox2*^Δ^. (B) *shox2*^Δ^ (Δ) and *shox2^+^* (+) PCR products using DNA obtained from *shox2^+^*^/+,^ *shox2*^Δ*/+,*^ and *shox2*^Δ*/*Δ^ embryos. (C) Quantification of *shox2* transcript levels by RT-qPCR. *shox2^+/+^* (1.00±.08; *n*=11), *shox2*^Δ*/+*^ (0.59±0.05; *n*=8) and *shox2*^Δ*/*Δ^ (0.0003±0.0001; *n*=11) embryos. Each dot represents data from an individual embryo at 24 hpf. (D) Section of the otic placode in 18 somite stage embryos after *in situ* hybridization with shox2 probe in *shox2^+/+^* and *shox2*^Δ*/*Δ^ embryos. Whole-mount immunofluorescence labeling of Shox2 on 24 hpf embryos from (E) *shox2^+/+^*, (F) *shox2*^Δ*/+*^ and (G) *shox2*^Δ*/*Δ^ larva in the *Tg(pax2a: GFP)* reporter background. Fluorescent images from *Tg (pax2a: GFP)* (green) reporter, Shox2 (red) immunostaining, merged image with Hoechst (blue). Magnified images show optical sections of merged images. Arrows point to the diencephalic region with Shox2 expression. Optical sections of the region flanked by d1 and d2 containing nuclear Shox2 labeling were used for fluorescence quantification. (H) Shox2 immunofluorescence signal from individual cells taken from *shox2^+/+^* (3544±44.8 a.u., *n*=965 cells, 12 embryos), *shox2*^Δ*/a*^ (2279±32.1 a.u., *n*= 687 cells, ten embryos) and *shox2*^Δ*/*Δ^ (744.9±4.9 a.u., *n*=885 cells, 11 embryos). A threshold of two standard deviation above average background signal (1053 a.u.) was set (dotted line). Cells above the threshold were considered Shox2 positive and used for comparison. (I) Percent of Shox2 expressing cells in cohort in *shox2^+/+^* (95.3±1.6%), *shox2*^Δ*/a*^ (83.2±3.9%) and *shox2*^Δ*/*Δ^ (1.95±1.0%) larvae. Cell and embryo numbers as listed above. One-way ANOVA and Sidak multiple comparisons tests were used for statistical analysis. Values reported as mean±s.e.m. **P≤0.01, ***P≤ 0.001; ****P≤ 0.0001. d1-d2; diencephalic domain 1 and 2; MBH, mid hindbrain boundary; a.u., arbitrary units, s.e.m. standard error of the mean. Scale bars: 10 µm.

To establish the function of *shox2* during otic development, a loss-of-function *shox2* mutant zebrafish line was generated using CRISPR/Cas9 genome editing. The homeobox domain in the Shox2 protein is essential for DNA binding and subsequent function of the protein to regulate transcription. Deletion of just the homeobox domain may lead to aberrant gene products that retain activity and act as dominant negatives. Instead, exons 1 and 2 of the *shox2* gene were targeted for deletion to ensure that transcription and translation of the protein is disrupted early in the coding sequence so that little to no protein would be made. Two distinct sgRNAs were designed to cleave within exon 1 and 2 (Fig. 1A). Repair and joining of the cleaved sites generated several *shox2* deletion alleles. One of the mutant alleles, *shox2*^Δ*a*^, hereafter referred to as *shox2*^Δ^, was identified and validated by Sanger sequencing. The shox2^Δ^ allele contains a ∼1.6 kilobase pairs (kb) deletion (Δ) of the predicted exons 1 and 2 regions in *shox2* and produced an early stop codon (Fig. 1A). To identify the genotype of zebrafish, primer pairs that anneal specifically to *shox2^+^*and *shox2*^Δ^ alleles were designed for PCR genotyping. Incrossing of *shox2*^Δ*/+*^ heterozygotes in a mixed genetic background produced a cohort of wild-type, heterozygote and mutant embryos as detected by PCR genotyping (Fig. 1B). The incrosses showed a near Mendelian ratio of wild-type (28.2±1.6%), heterozygote (50.7±2.7%) and mutant fish with a slight reduction in the percentage of *shox2*^Δ*/*Δ^ (21.1±4.8%) embryos that survived to 5 dpf. After allowing the cohort of fish to mature to 2 months of age, wild type and heterozygotes survived, but no adult mutant *shox2*^Δ*/*Δ^ fish could be identified (Fig. S2A). Incrosses of *shox2*^Δ*/+*^ heterozygotes in an isogenic background (EKW) only had wild-type (23.4±4.4%) and heterozygote (76.6±4.4%) fish at 5 dpf. At 2 months of age, wild-type and heterozygotes constituted the cohort (Fig. S2B). These results suggest that *shox2* is essential for embryonic development and survival of fish. Moreover, differences in embryonic survival in dissimilar genetic backgrounds suggest the potential presence of modifier genes.

To show that the *shox2*^Δ^ mutation results in loss of *shox2* mRNA transcript, cohorts of embryos were collected and individual embryos were subjected to RT-qPCR. Individual embryos were collected at 24 hpf, total RNA was extracted and used for cDNA synthesis. cDNA obtained from individual embryos were subjected to qPCR using primers that anneal to the *shox2* cDNA. Samples were first normalized to *eef1a1l1* and relative *shox2* transcript levels from samples were compared to *shox2* levels in wild-type embryos. Primers for RT-qPCR are listed in Table 1. *shox2* transcript in *shox2*^Δ*/+*^ is reduced approximately by half relative to *shox2^+/+^* embryos (*P*<0.001), while *shox2* transcript in homozygous mutant embryos were virtually eliminated compared to both *shox2^+/+^* (*P*<0.0001) and *shox2*^Δ*/+*^ (*P*<0.0001) (Fig. 1C).

**Table 1.**
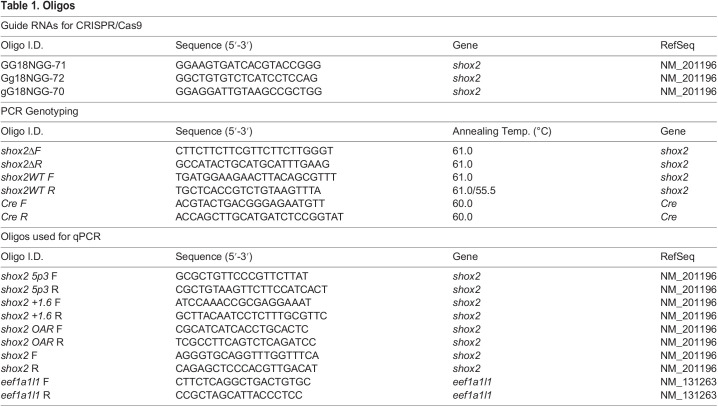
Oligos

To determine if the *shox2* transcript is absent from the otic placode, *shox2^+/+^* and *shox2*^Δ*/*Δ^ were harvested at the 18 somite stage and whole mount *in situ* hybridization performed. Sections of the embryos at the otic placode region were acquired and showed the presence of shox2 *in situ* signal at the otic placode in *shox2^+/+^* embryos but was not detected in the *shox2*^Δ*/*Δ^ embryos (Fig. 1D). To determine Shox2 protein expression levels, embryos were assessed by quantitative immunofluorescence. The diencephalon region in 24 hpf embryo was used for quantification due to the large number of cells expressing high levels of Shox2 protein. The *Tg (pax2a: GFP)* reporter was introduced to provide fluorescently labeled anatomical landmarks of the diencephalic domains 1 (d1) and 2 (d2) in order to identify and compare cells in the same region between fish of different genotypes (Picker et al., 2002). Cells expressing Shox2 protein in the developing diencephalon region flanked by d1 and d2 were detected in both *shox2^+/+^* (Fig. 1E) and *shox2*^Δ*/+*^ (Fig. 1F) but were markedly absent in *shox2*^Δ*/*Δ^ embryos (Fig. 1G). Nuclear fluorescence intensity of individual cells from the antibody labeling were used to quantify Shox2 protein levels in a cohort of embryos. Fluorescence intensity of Shox2 labeling in *Tg shox2*^Δ*/*Δ^ (744.9±4.9 a.u.) was significantly reduced when compared to either *shox2^+/+^* (3544±44.8 a.u., *P*<0.0001) or *shox2*^Δ*/+*^ (2279±32.1 a.u., *P*<0.001) embryos (Fig. 1H). Furthermore, the percentage of Shox2 expressing cells in *shox2*^Δ*/*Δ^ embryos was virtually eliminated (1.9±1.0%) when compared to *shox2^+/+^* embryos (95.3±1.6%, *P*<0.0001) and *shox2*^Δ*/+*^ embryos (83.2±3.9%, *P*<0.0001) (Fig. 1I). These data indicate that mutant *shox2*^Δ*/*Δ^ embryos lack both transcript and protein.

### shox2 null mutants display behavior associated to balance deficiencies

Hearing-specialized fish such as zebrafish use the saccule and lagena to mediate auditory function, while the utricle is used for balance (Riley and Phillips, 2003). The utricular and saccular maculae are innervated by SAG that delaminate from the anteroventral region of the otic vesicle (Haddon and Lewis, 1996). Lineage tracing showed several distinct populations of otic precursors in the developing zebrafish inner ear that become SAGs and hair cells (Sapede et al., 2012). Dye tracing experiments show two spatially segregated SAG neuronal populations, the anterior and posterior SAG innervates hair cells in the utricle and saccule, respectively (Sapede and Pujades, 2010). From our data, *shox2* expressing cells are initially detected in the otic placode where neurosensory precursors reside and continues to be expressed as precursors in the anteroventral region of the otic vesicle commit to become neuronal or sensory precursors (Sapede et al., 2012; Sapede and Pujades, 2010). To determine whether ablation of *shox2* affects balance or audition, we examined the anatomic development and performed behavioral testing on the larva.

**Fig. 2.**
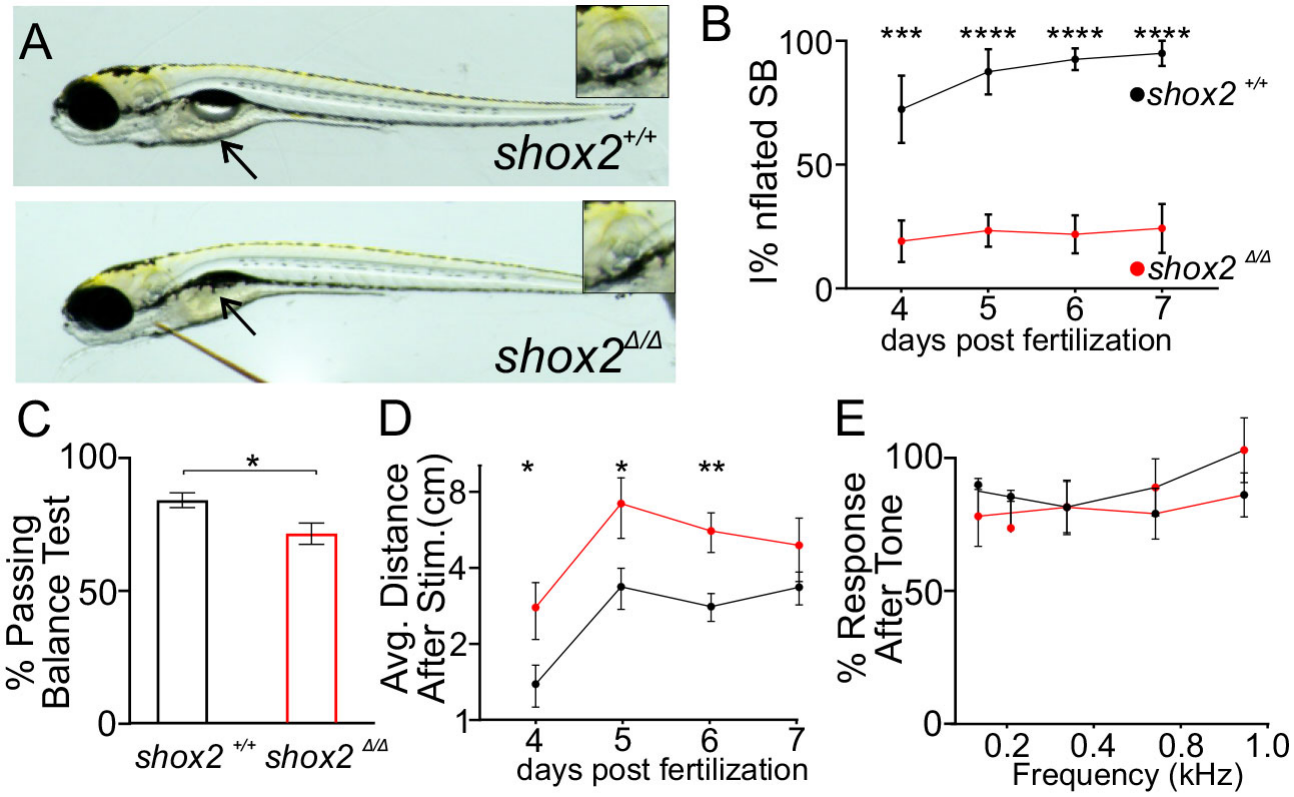
**Analyzing vestibular and auditory behavior of *shox2* mutant larvae.** (A) Representative images of *shox2^+/+^* and *shox2*^Δ*/*Δ^ 5 dpf zebrafish larvae. The arrow points to the swim bladder (SB). (B) Percent of embryos with inflated swim bladder at different ages in *shox2^+/+^* (4 dpf: 72.4±13.6%, 5 dpf: 87.5±9.2%, 6 dpf: 92.6±4.4%, 7 dpf: 95.0±5.1%), and *shox2*^Δ*/*Δ^ (4 dpf: 19.1±8.4%, 5 dpf: 23.4±6.5%, 6 dpf: 21.9±7.7%, 7 dpf: 24.3±9.9%) larvae. (C) Percent of *shox2^+/+^* (82.7±2.8%, *n*=42) and *shox2*^Δ*/*Δ^ (70.3±4.0%, *n*=37) larva passing the balance test (BT), embryos from different ages were combined. (D) Average distance traveled by *shox2^+/+^* (4 dpf: 1.4±0.3 cm, 5 dpf: 3.4±0.6 cm, 6 dpf: 2.8±0.4 cm, 7 dpf: 3.4±0.5 cm) or *shox2*^Δ*/*Δ^ (4 dpf: 2.8±0.7 cm, 5 dpf: 7.2±2.0 cm, 6 dpf: 5.6±1.0 cm, 7 dpf: 4.9±1.4 cm) larva after mechanical stimulation. (E) Percent of embryos responding to a series of tone pip in *shox2^+/+^* (*n*=15) and *shox2*^Δ*/*Δ^ (*n*=22) larvae. Tone bursts were presented at 0.1, 0.2, 0.3, 0.5 and 1 kHz and movements evoked from acoustic stimuli were recorded. Three independent trials were accomplished for each frequency and showed no significant (ns) differences. One-way ANOVA and Sidak multiple comparisons tests were used for statistical analysis. Values reported as mean±s.e.m. *P*≤0.05, **P≤0.01, ***P≤ 0.001; ****P≤ 0.0001. Values reported as mean±s.e.m.

During the larval period, zebrafish rely mostly on the utricle to detect and maintain posture (Bagnall and Schoppik, 2018; Riley and Moorman, 2000). At this developmental stage, semicircular canals do not significantly contribute to vestibular function (Lambert et al., 2008). To maintain balance and posture, the zebrafish larva counteracts gravity by inflating their swim bladders to remain buoyant (Bagnall and Schoppik, 2018). Utricular dysfunction in zebrafish results in an uninflated swim bladder (Kappler et al., 2004; Kwak et al., 2006; Riley and Grunwald, 1996; Riley and Moorman, 2000; Smith et al., 2020). The 5 dpf larvae were assessed for the presence of a swim bladder with examiners blinded to the genotype. The majority of *shox2*^Δ*/*Δ^ larvae did not have an inflated swim bladder despite the lack of gross morphological abnormalities (Fig. 2A). To determine if *shox2*^Δ*/*Δ^ larvae are delayed in swim bladder inflation, zebrafish larvae were scored daily for swim bladder inflation from 4 to 7 dpf (Fig. 2B). At 4 dpf, 72.4±13.6% of *shox2^+/+^* larvae had an inflated swim bladder. In contrast, only 19.1±8.4% (*P*<0.001) age-matched *shox2*^Δ*/*Δ^ larvae had inflated swim bladders. By 5 dpf, swim bladder inflation of *shox2^+/+^* increased to 87.5±9.2%, whereas the *shox2*^Δ*/*Δ^ population swim bladder inflation was at 23.4±6.5% (*P*<0.0001). At 6 and 7 dpf, the majority of *shox2^+/+^* larvae had an inflated swim bladder (6 dpf: 92.6±4.4% and 7 dpf: 95.0±5.1%), while low percentages of *shox2*^Δ*/*Δ^ larva have inflated swim bladders (6 dpf: 21.9±7.7%; *P*<0.0001 and 7 dpf: 24.3±9.9%, *P*<0.0001). These data suggests that *shox2*^Δ*/*Δ^ do not inflate their swim bladders potentially due to vestibular dysfunction.

The ability to maintain balance was assessed in 4-7 dpf *shox2^+/+^* and *shox2*^Δ*/*Δ^ larvae by observing the position of the fish after mechanical stimulation. The ability of the larvae to maintain an upright position after they have stopped moving was scored as passing the balance test, while fish that could not remain upright failed the balance test (Kwak et al., 2006; Riley and Moorman, 2000). All the *shox2* mutant zebrafish with uninflated swim bladders could not maintain balance. The remaining *shox2* mutant zebrafish that did have inflated swim bladders were assayed separately and the percentages of embryos that passed the balance test were averaged. Compared to their *shox2^+/+^* (82.7±2.8%) counterparts, we observed a statistically significant lower percentage of *shox2*^Δ*/*Δ^ (70.3±4.0%, *P*<0.05) larvae passing the balance test (Fig. 2C). These results suggest that shox2 mutant zebrafish cannot properly maintain balance even with an inflated swim bladder.

Zebrafish larvae utilize swim bouts to maintain balance and stability (Bagnall and Schoppik, 2018). As the larvae mature, swim bouts are triggered by unstable sensory input (Schoppik et al., 2017). Its inability to balance is reflected by the increased distance traveled during a given period. To measure the distance traveled in zebrafish larva swim bouts, a mechanical stimulus was applied to the caudal fin and the travel distance by the larvae was recorded for one minute. *shox2^+/+^* and *shox2*^Δ*/*Δ^ larva at four distinct ages (4-7 dpf) were recorded. From 4-6 dpf, *shox2*^Δ*/*Δ^ (4 dpf: 2.8±0.78 cm, *P*<0.05; 5 dpf: 7.2±2.0 cm, *P*<0.05; 6 dpf: 5.6±1.0 cm, *P*<0.01) larvae swam greater distances than the *shox2^+/+^* (4 dpf:1.4±0.3 cm, 5 dpf: 3.3±0.6 cm, 6 dpf: 2.8±0.4 cm) siblings. At 7 dpf *shox2*^Δ*/*Δ^ (4.9±1.4 cm), larva still traveled greater distances than *shox2^+/+^* (3.4±0.5 cm), but the difference was not statistically significant (Fig. 2D). Although significant differences in the average distance traveled was observed from 4-6 dpf, no significant differences were observed at 7 dpf. This could potentially be due to compensatory mechanisms for maintaining balance in the fish. Together the data suggests that *shox2*^Δ*/*Δ^ larva have mild vestibular deficits as shown by the inability to inflate their swim bladder and maintain balance at early larval stages.

Next, we sought to determine if auditory function was perturbed in *shox2* deficient larvae. Acoustic stimuli are mostly detected by the saccule. Frequencies at 0.1-1.0 KHz can be detected by zebrafish within the first week of development (Bhandiwad et al., 2013; Zeddies and Fay, 2005) To initiate an acoustic startle response, zebrafish were presented with a 0.1 s tone burst at different sound frequencies (0.1, 0.2, 0.3, 0.5 and 1.0 kHz). The response of larvae to acoustic stimulus was recorded and analyzed. The percentage of larvae that moved within 100 ms after the end of the tone was scored. Similar percentages of both *shox2^+/+^* (85.8, 83.3, 95.8, 76.4, 80.6%) and *shox2*^Δ*/*Δ^ (77.6, 91.9, 80.4, 72.4, 93.3%) larva responded to the respective frequency tones. The results suggest that *shox2*^Δ*/*Δ^ larvae can respond to sounds in a similar manner to wild-type larvae (Fig. 2E). Together, the behavioral data suggests that ablation of *shox2* results in vestibular but not auditory dysfunction early in development.

### shox2 is expressed in developing anterior SAG neurons

Next, we established *shox2* expression in inner ear cell types found in the anteroventral region of the otic vesicle during neurogenesis. Identifying cell types in developing anterior SAG neurons was accomplished by using *shox2* reporter lines and comparing fluorescent cells to other fluorescent reporters that label distinct cell types during otic development. The *shox2* reporter fish employs a bipartite *GAL4:UAS* expression system where the *GAL4* gene is driven by the endogenous *shox2* promoter (*shox2^Gal4^*). The *shox2*^Gal4^ knock-in transgenic zebrafish was generated using a single guide RNA (sgRNA) that targeted a region upstream of the *shox2* coding sequence. CRISPR/Cas9 mediated insertion of a donor plasmid containing a basal heat-shock promoter along with the GAL4 reporter gene (Kimura et al., 2014). Pairing *shox2^Gal4^* with an upstream activation sequence (UAS) driving genes coding fluorescent proteins allows GAL4 binding to the UAS and subsequent expression of genetically encodable fluorescent proteins such as EGFP (*UAS: EGFP*) or mCherry (*UAS: mCherry*). The *Tg (shox2^Gal4^, UAS: mCherry) and Tg (shox2^Gal4^, UAS: GFP) shox2* transgenic reporter lines were introduced to different fluorescent reporters that label otic cell types and developing inner ear neurons. Intersectional labeling of cells from the *shox2* reporter and fluorescent reporter helped to identify cell types that express *shox2*.

**Fig. 3.**
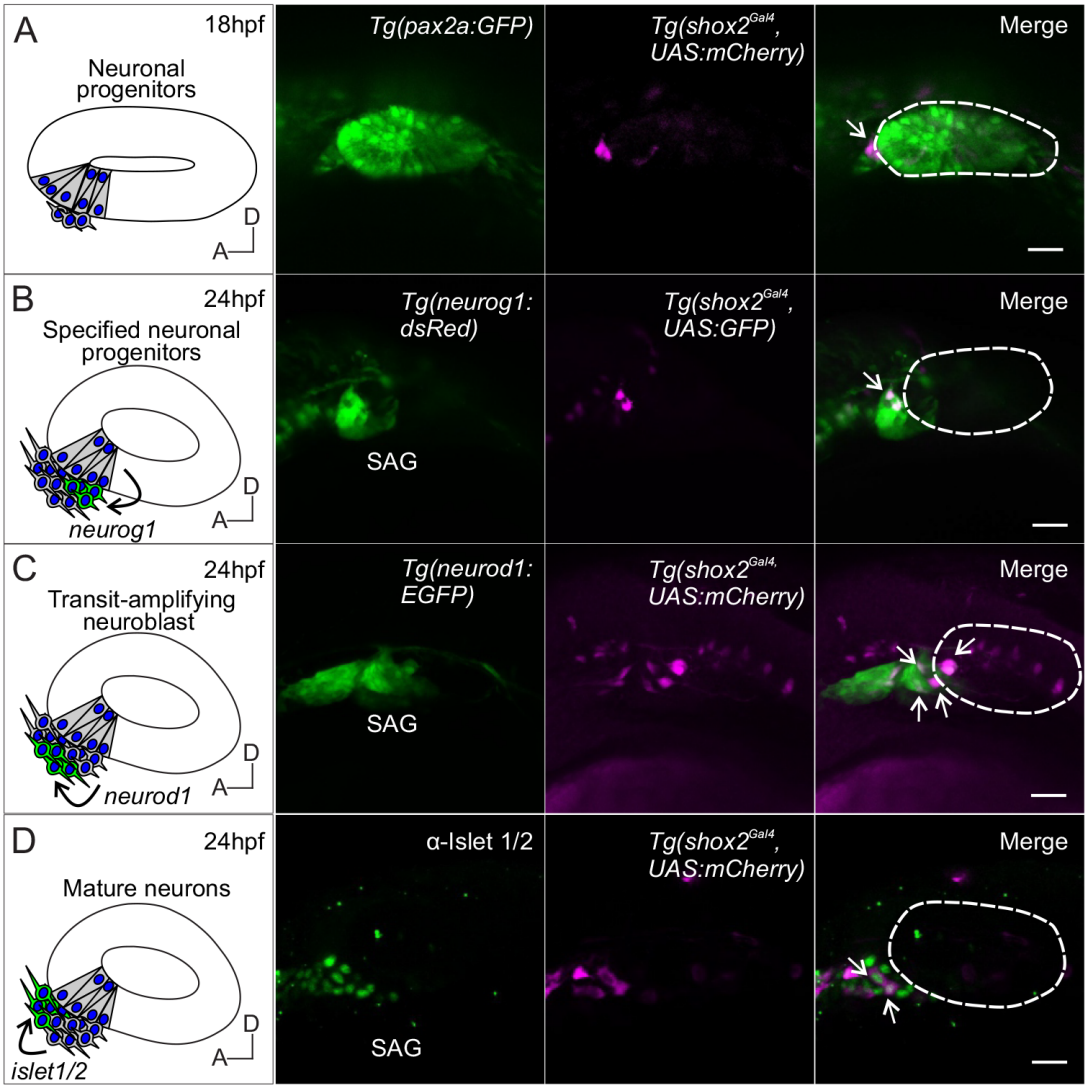
**Lineage labeling of neurogenic cells in the developing SAG using *shox2* fluorescent reporter.** Fluorescent images of developing inner ear from *Tg(shox2^Gal4^,UAS:GFP)* reporter larva with inner ear and neuronal reporters. Curved arrows on diagrams indicate migration of developing neurons from its previous locations. Dashed lines in merged images outline the otic vesicle. (A) Diagram of neuronal progenitors in the anteroventral region of the otic vesicle at 18 hpf. Reporter fluorescence from a pan-otic *Tg(pax2a: GFP)* and *Tg (shox2^Gal4^,UAS:mCherry)* at 18 hpf. Merged image depicts *shox2* reporter labeled cells (arrow) in the anteroventral region adjacent to the otic vesicle (*n*= 5). (B) Diagram of delaminating neuroblasts expressing *neurog1* (green) at the floor of the otic vesicle. Fluorescence from Tg*(neurog1: dsRed)* and *Tg(shox2^Gal4^,UAS:GFP)* reporters at 24 hpf. Merged image identifies specified neuroblasts and delaminating neuroblasts (arrows) (*n*=14 embryos). (C) Diagram of transit-amplifying neuroblasts expressing *neurod1* (green) at 24 hpf after delaminating from the otic vesicle. Fluorescence from *TgBAC(neurod1: EGFP)* and *Tg(shox2^Gal4^,UAS:mCherry)* reporters at 24 hpf. Merged image shows overlap of *neurod1* and *shox2* reporter fluorescence (arrows) (*n*=9 embryos). (D) Diagram of mature neurons expressing Islet 1/2 (green) from transit amplifying neuroblasts (arrow). Fluorescence from the medial position of the neurogenic domain shows Islet 1/2 immunostaining and *Tg(shox2^Gal4^, UAS:mCherry)* reporter at 24 hpf. Merged image shows overlap of Islet 1/2 and *shox2* reporter marked cells (arrow) (*n*= 11 embryos). SAG, statoacoustic ganglion. Scale bars: 10 µm.

At 18 hpf, the neuronal and sensory progenitors are in the anteroventral region of the otic vesicle. To mark cells in the otic vesicle at this developmental time, a *Tg (pax2a:GFP)* (green) reporter fish was used (Picker et al., 2002). *shox2* reporter fish were introduced into the pan otic reporter and co-labeling of cells compared to identify cell types*.* GFP labeled cells from the *pax2a* reporter marked the otic vesicle. mCherry expressing cells from the *shox2* reporter were located outside the otic vesicle in the anteroventral region corresponding to delaminating neuronal progenitors (Fig. 3A).

To determine if *shox2* expression was present later in SAG development, fluorescent images were acquired from reporter fish at 24 hpf. At this time point, specified and delaminating neuronal progenitors, transit-amplifying neuroblasts and mature neurons are present (Vemaraju et al., 2012). We identified *shox2* expression in delaminating neuroblast at 24 hpf embryos with the *shox2* reporter *Tg (shox2^Gal4^, UAS:GFP)* (magenta) and a neuronal progenitor reporter *Tg (neurog1: dsRed)* (green)*.* GFP expressing cells from the *shox2* reporter overlapped with dsRed marked delaminated neuroblasts outside the anteroventral region of the otic vesicle (Fig. 3B). To track the expression of *shox2* in transit-amplifying neuroblasts, fluorescent images from embryos with the *shox2* reporter *Tg(shox2^Gal4^, UAS:mCherry)* (magenta) and *Tg (neurod1:EGFP)* (green) were acquired. mCherry expressing cells overlap with EGFP expressing neuroblasts (Fig. 3C). Mature neurons from the *shox2* reporter were detected by immunolabeling of Isl1/Isl2b (green) in Tg *(shox2^Gal4^, UAS: mCherry)* (magenta) embryos. Nuclear expression of Isl1/Isl2b protein in mCherry expressing cells at 24 hpf in the SAG region (Fig. 3D). At 48 hpf, when the vast majority of cells in the anterior SAG are mature neurons, the *shox2* reporter signal is no longer present in the inner ear (Fig. S3). The reporter analysis shows that *shox2* is expressed in otic neuronal progenitors at the anterior region of the otic vesicle, maintains its presence in neuroblasts and nascent neurons at 24 hpf, but is no longer expressed at 48 hpf in mature neurons.

### shox2 ablation decreases anterior SAG neuron numbers

**Fig. 4.**
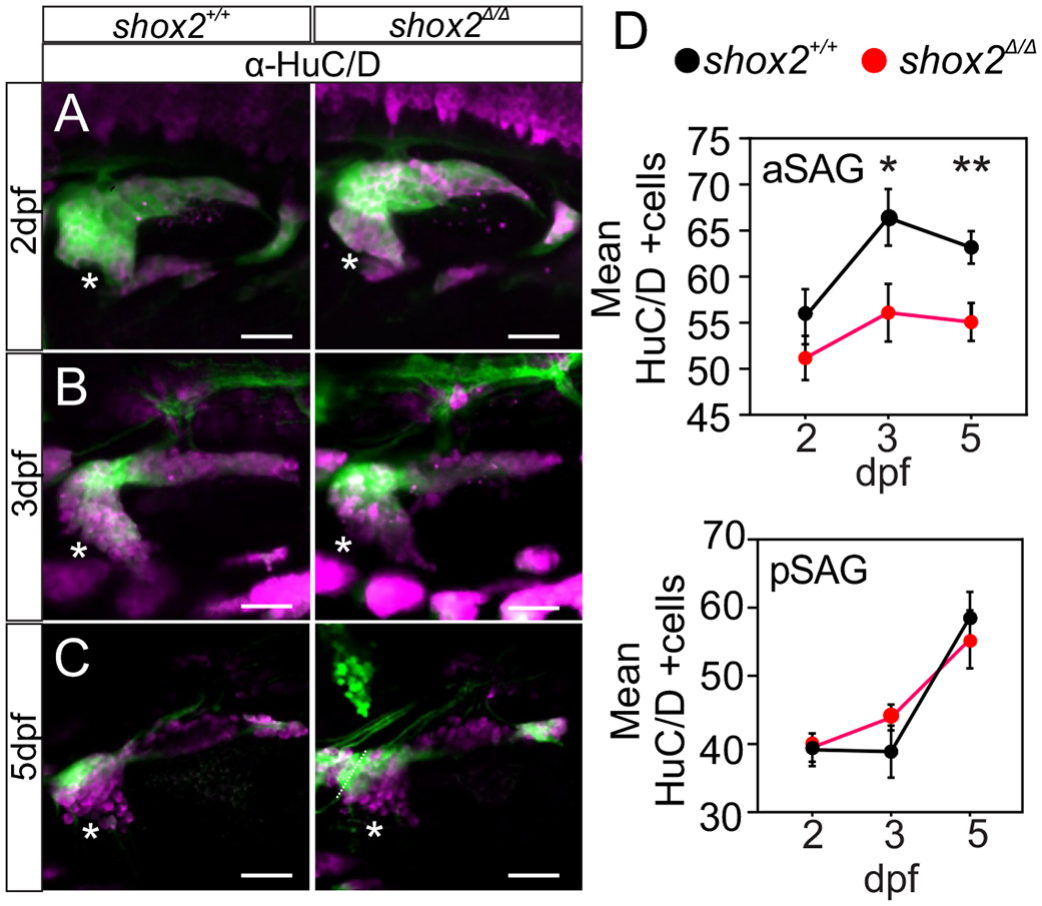
**Comparing number of SAG between *shox2^+/+^* and *shox2^Δ/Δ^* animals.** HuC/D immunostaining of *shox2^+/+^* and *shox2^Δ/Δ^* larvae that contain the *Tg(neurod1: EGFP)* reporter at (A) 2, (B) 3 and (C) 5 dpf. EGFP (green) and HuC/D (magenta) are shown. Asterisks mark the anterior SAG (aSAG). (D) HuC/D cell counts from aSAG are as follows: 2 dpf, *shox2^+/+^* (55.7±3.0 cells, *n*=12 larvae), *shox2^Δ/Δ^* (51.2±2.4 cells, *n*=11 larvae), 3 dpf, *shox2^+/+^* (66.4±3.1 cells, *n*=10 larvae), *shox2^Δ/Δ^* (56.1±3.1 cells, *n*=11 larvae), 5 dpf, *shox2^+/+^* (63.2±1.8 cells, *n*=11 larvae), *shox2^Δ/Δ^* (55.1±2.1 cells, *n*=12 larvae). (E) HuC/D cell counts from posteromedial SAG (pSAG) are as follows: 2 dpf, *shox2^+/+^* (39.5±1.9 cells, *n*=15 larvae), *shox2^Δ/Δ^* (39.1±1.8 cells, *n*=14 larvae), 3 dpf, *shox2^+/+^* (38.9±3.8 cells, *n*=10 larvae), *shox2^Δ/Δ^* (43.9±1.9 cells, *n*=11 larvae), 5 dpf, *shox2^+/+^* (58.6±3.7 cells, *n*=13 larvae), *shox2^Δ/Δ^* (55.4±4.3 cells, *n*=14 larvae). One-way ANOVA and Sidak multiple comparison test were used. *P≤0.05, **P≤0.01. Values reported as mean±s.e.m. Scale bars: 10 µm.

**Fig. 5.**
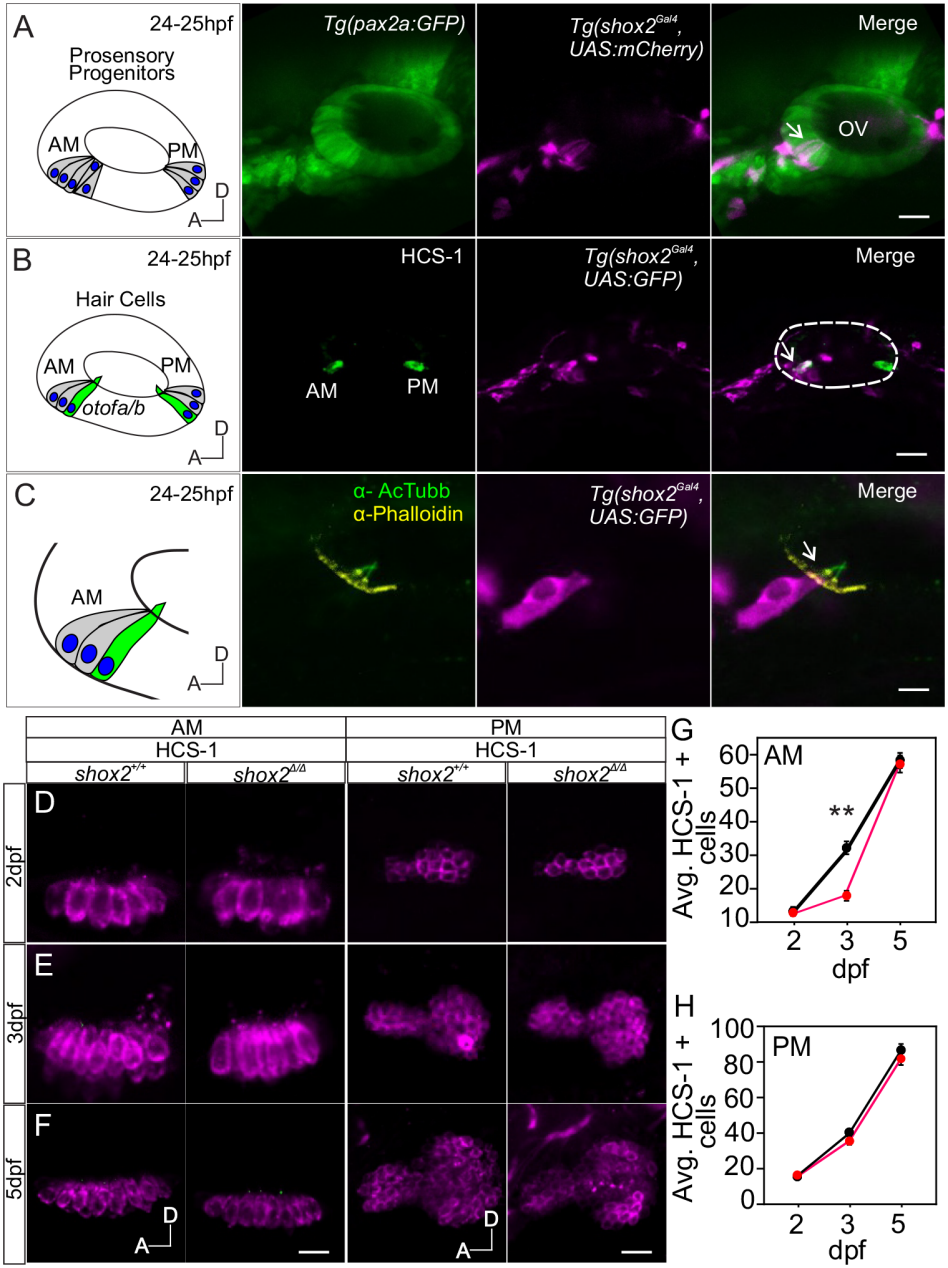
**Comparing number of hair cells between *shox2^+/+^* and *shox2^Δ/Δ^* animals.** (A) Diagram depicting the developing sensory domain (gray) in the inner ear at 24-25 hpf with developing hair cells expressing otoferlin a and b (otof a/b) in the anterior macula (AM) and posterior macula (PM). Fluorescent images of Tg (shox2^Gal4^, UAS:mCherry) reporter with HCS1 antibody immunofluorescence labeling at 24-25 hpf. The HCS-1 antibody recognizes Otoferlin. The merged image depicts *shox2* reporter labeled cells (arrows) within the OV (otic vesicle) in the anteroventral region (*n*=9 embryos). Dashed lines mark the otic vesicle. Whole-mount HCS-1 immunolabeling of hair cells from the AM and PM in *shox2^+/+^* and *shox2^Δ/Δ^* larvae at (B) 2, (C) 3 and (D) 5 dpf were acquired for quantification. (E) HCS-1 cell counts from the AM are as follows: 2 dpf, *shox2^+/+^* (12.9±0.8 cells, *n*=18 larvae), *shox2^Δ/Δ^* (12.8±1.0 cells, *n*=18 larvae), 3 dpf, *shox2^+/+^* (28.3±1.6 cells, *n*=12 larvae), *shox2^Δ/Δ^* (20.7±1.5 cells, *n*=13 larvae), 5 dpf, *shox2^+/+^* (57.2±1.8 cells, *n*=16 larvae), *shox2^Δ/Δ^* (55.2±1.8 cells, *n*=15 larvae). (F) HCS-1 cell counts from the PM are as follows: 2 dpf, shox2^+/+^ (18.7±1.3 cells, *n*=13 larvae), shox2^Δ/Δ^ (18.9±1.0 cells, *n*=13 larvae), 3 dpf, shox2^+/+^ (41.6±1.2 cells, *n*=12 larvae), shox2^Δ/Δ^ (37.3±2.1 cells, *n*=12 larvae), 5 dpf, shox2^+/+^ (87.1±3.2 cells, *n*=19), shox2^Δ/Δ^ (80.0±2.9 cells, *n*=20 larvae). One-way ANOVA and Sidak multiple comparison test were used. **P≤0.01. Values reported as mean±s.e.m. Scale bars: 10 µm.

Considering balance deficiencies in *shox2*^Δ*/*Δ^ larva and the presence of shox2 reporter signal in neuronal progenitors, we wanted to determine whether *shox2* affects development of anterior SAG neurons that innervate the utricle and spares the posterior SAG neurons that innervate the saccule. The anterior and posterior SAG neurons can be distinguished by their anatomical location (Sapede and Pujades, 2010). We exploited the spatial segregation of the anterior and posterior SAGs to ascertain whether absence of *shox2* affects development of the two population of neurons. To aid in identifying the anterior and posterior SAG, the *Tg (neurod1: EGFP)* reporter was used for fate mapping of developing neurons. To mark nascent post-mitotic neurons, the HuC/D antibody was used (Andermann et al., 2002). The number of neurons in *shox2^+/+^* and *shox2*^Δ*/*Δ^ larva were assessed at 2, 3, and 5 dpf (Fig. 4A-C). Comparing the number of anterior SAG neurons from *shox2^+/+^* and *shox2*^Δ*/*Δ^ animals revealed a decrease in neuronal numbers starting at 2 dpf (*shox2^+/+^*: 55.7±3.0 cells, *shox2*^Δ*/*Δ^: 51.2±2.4 cells). As development proceeded, a statistically significantly drop in the number of anterior SAG at 3 dpf (*shox2^+/+^*: 66.4±3.1 cells, *shox2*^Δ*/*Δ^: 56.1±3.1 cells, *P*<0.05) and 5 dpf (*shox2^+/+^*: 63.2±1.8 cells, *shox2*^Δ*/*Δ^: 55.1±2.1, *P*<0.01) was observed (Fig. 4D). Cell counts in the pSAG showed that neuronal numbers were similar between wild-type and mutant animals (Fig. 5E). The data indicates that absence of *shox2* decreases the number of anterior SAG neurons that innervate the utricle but retains the same numbers of posterior SAG neurons. The findings are consistent with the observed vestibular deficiencies in *shox2*^Δ*/*Δ^ and suggests that *shox2*^Δ*/*Δ^ fish lack a full complement of anterior SAGs.

### shox2 is expressed in developing hair cells but shox2 ablation does not affect hair cell numbers

The neurogenic region is adjacent to the prosensory progenitor domain that gives rise to hair cells in the anterior maculae of the otic vesicle. Using double transgenic embryos harboring the pan-otic Tg(*pax2a:GFP)* (green) and *shox2* Tg(*shox2^Gal4^, UAS: mCherry*) (magenta) reporters, the presence of shox2 reporter cells was observed in the sensory epithelium at 24-25 hpf (Fig. 5A, arrow)**.**

Cells in the prosensory domain give rise to hair cells and supporting cells in the anterior maculae (Haddon and Lewis, 1996; Riley and Grunwald, 1996). At 24 hpf, a small number of cells, called tether cells, precociously develop into the first HCs of the anterior maculae. As the tether cells mature, they express hair cell markers such as otoferlin (Chatterjee et al., 2015; Riley and Grunwald, 1996). To ascertain if *shox2* expressing cells observed in this domain are tether cells, *shox2* reporter embryos, *Tg (shox2^Gal4^, UAS: GFP)* were subjected to immunofluorescence using an antibody against hair cell soma 1 (HSC-1) that detects otoferlin proteins (Chatterjee et al., 2015). Immunofluorescence labeling using the HSC-1 antibody co-localizes with *shox2* expressing cells in the anterior maculae. Furthermore, *shox2* expressing cells show acetylated tubulin labeled kinocilium (green) and phalloidin labeling of the cuticular plate and stereocilia (yellow) (Fig. 5C, arrow). The data suggests that *shox2* expression is initially detected in sensory precursors that develop into hair cells in the anterior maculae.

The expression of the *shox2-*reporter showed that *shox2* was present in early-born hair cells in the anterior maculae. To determine whether hair cell numbers were altered in the absence of *shox2*, hair cells counts were done in *shox2^+/+^* and *shox2*^Δ*/*Δ^ larvae labeled with HCS-1 antibody. Images of hair cells from the anterior and posterior macula at 2, 3 and 5 dpf were acquired and used for quantification (Fig. 5D-F). In the anterior maculae, at 2 dpf no significant change in average hair cell numbers for individual larvae was observed (*shox2^+/+^*: 12.9±0.8 cells, *shox2*^Δ*/*Δ^: 12.8±1.0 cells). At 3 dpf, hair cell numbers were significantly decreased in *shox2*^Δ*/*Δ^ compared to control (*shox2^+/+^*: 28.3±1.6 cells, *shox2*^Δ*/*Δ^: 20.7±1.5 cells, *P*<0.01). However, by 5 dpf, the number of hair cells in *shox2*^Δ*/*Δ^ recovered and was comparable to control (*shox2^+/+^*:57.2±1.8 cells, *shox2*^Δ*/*Δ^: 55.2±1.8 cells) (Fig. 5G).

Since no *shox2* expressing cells were observed in the posterior maculae, the number of hair cells in the posterior maculae should not change after *shox2* ablation. The hair cell numbers in the posterior maculae were used as controls. As development proceeded, the average number of hair cells in individual larvae increased from 2 dpf (*shox2^+/+^*: 18.7±1.3 cells, *shox2*^Δ*/*Δ^: 18.9±1.0 cells), 3 dpf (*shox2^+/+^*: 41.6±1.2 cells, *shox2*^Δ*/*Δ^: 37.3±2.1 cells) and 5 dpf (*shox2^+/+^*: 87.1±3.2 cells, *shox2*^Δ*/*Δ^: 80.0±2.9 cells), but no significant differences was observed after shox2 deletion in the posterior maculae (Fig. 5H). Overall hair cell numbers in either the anterior or posterior maculae were not significantly different in larvae by 5 dpf. The transient decrease in hair cell numbers in the anterior maculae observed in 3 dpf *shox2*^Δ*/*Δ^ may be confounded by both the small number of sensory progenitors expressing shox2 or by hair cell regeneration in zebrafish.

### Interrogating changes in otic cell progenitors by scRNA-seq

The above findings suggests that *shox2* is initially present in otic neurosensory progenitors during development and persists in prosensory and neuronal progenitors. Deletion of *shox2* decreases the neuronal population of anterior SAG neurons but do not significantly affect hair cell numbers in the anterior maculae. To determine how absence of *shox2* alters the molecular profile of cells undergoing SAG development, scRNA-seq was employed. This allows us to define otic cell types within a diverse cell population based on their transcriptome profile. By exploiting the asynchronous development of SAGs, we can also identify otic progenitors and developing cell types that arise from these cells. Analyzing scRNA-seq data allows classifications of different subpopulations of cells during development and allows us to interrogate molecular changes due to deletion of *shox2*. Since we observed a decrease in anterior SAGs, we focused first identifying the otic population of interest and performing analysis probing molecular changes only on otic cell types during inner ear neurogenesis. We obtained cells of the otic vesicle region from wild-type and mutant tissue from *shox2^+/+^*and *shox2*^Δ*/*Δ^ zebrafish embryos at 16-22 hpf, the period when otic precursors that are developing into post-mitotic neurons.

To facilitate fate analysis, *Tg (neurog1: dsRed, neurod1:EGFP), shox2^+/+^* double-positive embryos were harvested and dissociated into single cells for wild-type controls. The *neurog1* reporter is initially expressed in otic neuronal progenitors that reside within the otic vesicle, while the *neurod1* reporter is expressed in delaminating neuroblasts from the otic vesicle, transit-amplifying cells and nascent neurons (Hoijman et al., 2017). To obtain samples lacking *shox2*, Tg (*shox2^Gal4/^*^Δ^*, UAS:GFP*) fish were incrossed. This cross yields *shox2*^Δ*/*Δ^ non-fluorescent embryos that can be separated from GFP expressing embryos [Tg (*shox2^Gal4/Gal4^, UAS:GFP*)*, Tg*(*shox2^Gal4/^*^Δ^*, UAS:GFP*]. Individual fish from wild-type (GFP and dsRed positive) and mutant (GFP negative) cohorts were identified based on lack of fluorescence and genotyped. Tissues from around the otic vesicle region were dissected, while avoiding the hindbrain region and combined according to their genotypes. Tissues were dissociated into individual cells and subjected to the formation of gel beads in emulsion (GEMs). Individual cells from GEMs contained a library barcode to identify the cohort, a cell barcode to identify individual cells, and a unique molecular identifier (UMI) to identify unique molecules from each transcript. Reverse transcription in GEMs produces cDNA from individual cells that are marked by barcodes. From the scRNA-seq data, 10,466 wild-type cells with 94,679 mean reads/cell and 3165 median genes/cell were identified, while 8877 shox2^Δ/Δ^ cells with 102,922 mean reads/cell with 2946 median genes/cell were ascertained. scRNA-seq data from wild-type and mutant cells were aggregated together and used for analysis.

**Fig. 6.**
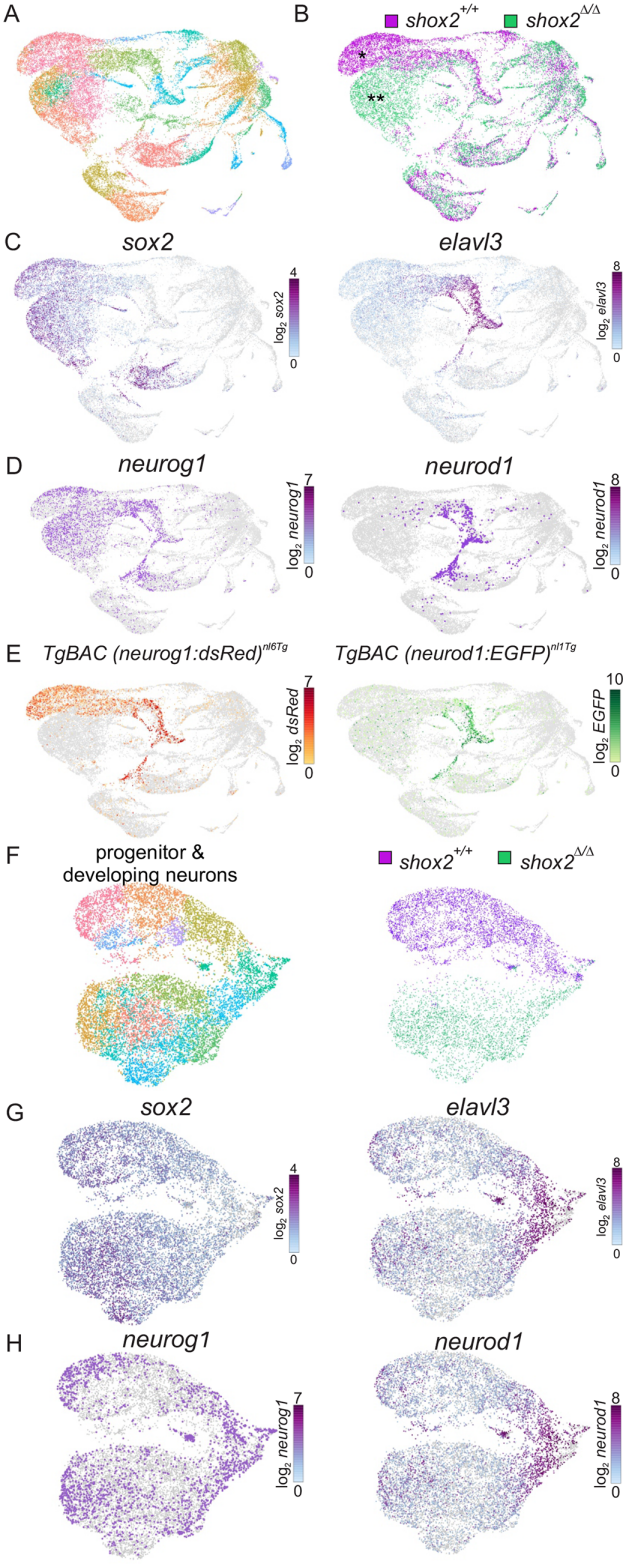
**Identifying cell populations in *shox2^+/+^* and *shox2*^Δ*/*Δ^ embryos by single cell RNA-seq.** (A) *shox2^+/+^* (*n*=59 embryos) and *shox2*^Δ*/*Δ^ embryos (*n*=43 embryos) at 16-22 hpf were harvested and tissue around the otic vesicle microdissected. Samples of the same genotype were pooled and subjected to scRNA-seq. Transcriptome of individual cells were aggregated, dimensionality reduction performed followed by unsupervised clustering before visualizing as a UMAP projection. Course-grain visualization of 29,152 cells identified 28 distinct cell types after clustering. (B) Cells from *shox2^+/+^* (magenta) and *shox2*^Δ*/*Δ^ (green) were identified using by library barcodes to display different and common cell populations. Major differences between wild-type and mutant cell populations were marked by single or double asterisks respectively. (C) *sox2* highlight progenitor cells while *elavl3* mark developing neurons. (D) *neurog1* and *neruod1* identify neuronal progenitors and developing neurons. (E) Cells expressing dsRed and EGFP from transgenes introduced into *shox2^+/+^* embryos. Cell clusters identified from graph-based clustering that overlapped with dsRed and EGFP in shox2^+/+^ and shox2^Δ/Δ^ were used to identify progenitors and developing neurons. (F) Plot of progenitor and development obtained from identified subset of cell clusters were obtained for further analysis. Progenitor and developing neuron populations from *shox2^+/+^* (magenta) and *shox2*^Δ*/*Δ^ (green) cells were identified using library identification barcodes for each genotype. (G) Sub-population of cells express *sox2* and *elavl3* to mark progenitor and developing neurons, respectively. (H) Sub- population of cells express *neurog1* and *neruod1* to mark neuronal progenitors and developing neurons, respectively.

To define the different cell types obtained from wild-type and mutant tissues, cell clustering was performed using the transcriptome of each cell. Dimensionality reduction and UMAP clustering of scRNA-seq from *shox2^+/+^* and *shox2*^Δ*/*Δ^ results display 28 distinct sub-population of cells from the otic vesicle region in the coarse-grain plot (Fig. 6A). To identify differences in the populations of cells, wild-type (magenta) to the mutant (green) cells were visualized on a UMAP plot based on their library barcode. Several cell populations displayed differences in UMAP coordinates suggestive of changes in cell identities that correspond to the ablation of *shox2* (Fig. 6B). The distinguishing gene expression features that define the different cell populations were identified and individual markers were surveyed. The population of cells that showed differences between wild-type and *shox2* mutant cells express *sox2* and *elavl3* (Fig. 6C). In zebrafish, both *sox2* and *elavl3* are expressed early during otic development. *sox2* establishes sensory competence in the inner ear (Gou et al., 2018) and elavl3 whose protein product is recognized by the HuC/D antibody labels developing SAG neurons (Andermann et al., 2002). The population overlaps with *neurog1* and *neurod1*, markers for neuronal progenitors and developing neurons, respectively (Fig. 6D), these cells correspond well to the dsRed and EGFP transcripts observed in the shox2^+/+^ cells (Fig. 6E). The cells that display differences are non-fluorescent cells obtained from mutant tissue that correspond to different types of progenitors and developing neurons whose transcriptomes have been altered in the absence of *shox2*. To further refine cells associated with the inner ear, sub-cluster analysis was employed to identify the otic population.

### Identifying altered otic progenitor populations in shox2 mutants

To identified *shox2^+/+^* and *shox2*^Δ*/*Δ^ progenitors and developing neurons, cell clusters that intersected with dsRed and EGFP were defined. Many of these cell clusters showed a bi-lobed distribution. Each lobe corresponded to *shox2^+/+^* or *shox2*^Δ*/*Δ^ cells. These *shox2^+/+^* and *shox2*^Δ*/*Δ^ cells within a cluster were different at the transcriptome level and showed separation in UMAP space, but were still similar enough to be classified as a distinct cell cluster (Fig. S4A). The identified individual cell clusters corresponding to progenitors and developing neurons were aggregated together for analysis (Fig. S4B). The *shox2^+/+^* and *shox2*^Δ*/*Δ^ showed the expected overlap of dsRed (Fig. S4C) and EGFP (Fig. S4D) expression in wild-type cells but not mutant cells in the identified cell clusters. By employing this strategy, cells from individual clusters were extracted using cell barcodes and reanalyzed to ascertain detailed differences. The re-clustered subpopulation of cells was displayed based on their genotype. Similar to the course-grain plot, the subpopulation of cells displayed multiple cell populations of progenitors and developing neurons that are distinct from each other due to *shox2* ablation (Fig. 6F). These cells showed *sox2* and *elavl3* expression (Fig. 6G) as well as *neurog1* and *neurod1* expression (Fig. 6H). These cells correspond to different progenitor and developing neurons obtained from *shox2^+/+^* and *shox2*^Δ*/*Δ^ tissues.

In this sub-population of cells, there are observable transcriptome differences between progenitor and developing neurons from *shox2^+/+^* and *shox2*^Δ*/*Δ^ tissue. To focus on the initial defect caused by shox2 ablation, we wanted to look at the differences between progenitors with the notion that any alteration in neurons likely originated from the initial improper establishment of progenitors.

During otic development, both intrinsic and extrinsic cues may affect development of progenitor cells. In otic progenitors, *shox2* may directly affect development of vestibular neurons. In contrast, *shox2* may also affect rhombomeres development and indirectly affect vestibular neuron development. Rhombomeres are a transient segment of the developing neural tube and are known to play an indirect role in otic development (Fekete, 1999). To identify otic and rhombomere progenitors, we used a set of previously identified markers that correspond to progenitors from the developing inner ear and rhombomere 5-7 (Tambalo et al., 2020).

**Fig. 7.**
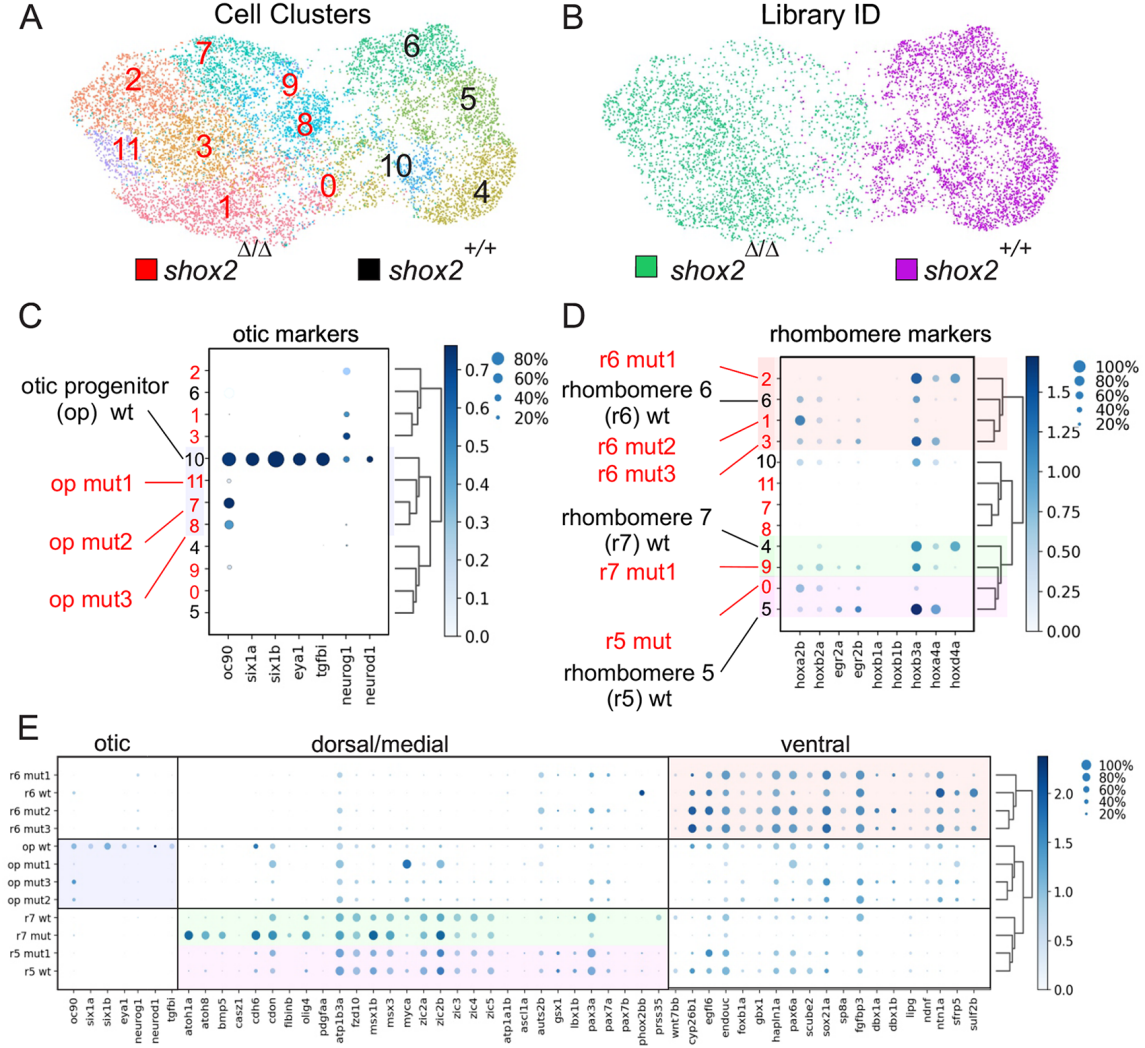
**Identifying progenitor populations from *shox2^+/+^* and *shox2*^Δ*/*Δ^ embryos.** Cell clusters that correspond to progenitors from the course-grain map were bioinformatically extracted and reanalyzed by unsupervised clustering before visualizing on UMAP coordinates. (A) Plot of progenitor populations from *shox2^+/+^* (magenta) and *shox2*^Δ*/*Δ^ (green) cells were identified using library identification barcode for each cell type. (B) Fine-grain UMAP plot shows 12 distinct cell clusters labeled from 0-11. *shox2^+/+^* clusters are labeled in black and *shox2*^Δ*/*Δ^ in red numerals. Dot plots were used to display marker gene expression. Each dot represents two values, the color of the dot represents relative gene expression levels and the size of the dot represents the percentage of cells expressing the gene. Hierarchical clustering identified related cell populations and the dendrogram represents the relationship between cell clusters. *shox2^+/+^* and *shox2*^Δ*/*Δ^ cell clusters are marked by black and red numbers, respectively. (C) Identifying otic progenitor (op) population that express inner ear marker genes (*oc90, six1a, six1b, eya1* and *neurog1*) were used to identify wild-type otic progenitor population. Hierarchical clustering revealed otic progenitors from wild-type and mutant cells that are highlighted by a blue box. (D) Identifying rhombomere cell populations expressing different *hox* genes (*hoxa2b*, *hox2a*, *egr2a*, *egr2b*, *hoxb1a*, *hox1b*, *hoxb3a*, *hoxa4a* and *hoxd4a*). Cell clusters were subjected to hierarchical clustering to identify cells from rhombomere (r) 5, 6 and 7. Cell populations from wild-type and mutant cells that are shaded in purple, red and green. (E) Expression of otic and developing rhombomere marker genes in progenitor populations. Dendrogram displays the relationship between clusters. Otic progenitor cluster are highlighted in blue, rhombomere 5, 6 and 7 clusters are highlighted in purple, red and green box, respectively.

Cell clusters with *neurog1* but not *neurod1* expression were identified as progenitors. These clusters were extracted using cell barcodes and subjected to re-clustering and analysis. Cell clusters from *shox2^+/+^* and *shox2*^Δ*/*Δ^ in the fine-grain UMAP plot displayed four sub-populations in *shox2^+/+^* embryos (clusters 4,5,6,10) while eight subpopulations of progenitors were identified in *shox2*^Δ*/*Δ^ (clusters 0,1,2,3,7,8,9,11) (Fig. 7A). These cell clusters remain distinctly segregated based on their genotype (Fig. 7B). To determine the identity of otic and rhombomere progenitors as well as the differences between *shox2^+/+^* and *shox2*^Δ*/*Δ^ progenitors, hierarchical clustering and marker gene expression was done. Hierarchical clustering using the cell transcriptome provides a dendrogram that displays the relationship of cell clusters, while marker gene expression shows key genes normally attributed to a specific cell population. To visualize relative marker gene expression, a dot plot was used. The detection rate and average gene expression cluster clusters were depicted. Darker colors indicate higher gene expression while the size of the larger dot indicates the detected proportion of cells from the cluster. Otic markers defined a cluster of cells that correspond to otic progenitors (op) in *shox2^+/+^* embryos and hierarchical clustering revealed three closely related clusters in *shox2*^Δ*/*Δ^ embryos (Fig. 7C). The wild-type otic cells expressed *oc90* (otoconin 90), a gene that codes for a major protein of the otoconia, a calcium carbonate structure in the saccule and utricle of the ear and serves as a scaffold for otoconia biomineralization (Wang et al., 1998). The inner ear cell types were also defined by the presence of *six1a*, *six1b*, *eya1* and *neurog1* (Tambalo et al., 2020). These genes code for transcription factors involved in specifying neuronal and sensory otic cell fate (Bricaud and Collazo, 2006; Wong et al., 2013), while the mutant otic progenitors have altered expression of these marker genes.

In addition to cell autonomous changes, development of the otocyst requires signals from the hindbrain. Mutations that affect patterning of the adjacent rhombomere regions can cause profound defects in the inner ear development (Fekete, 1999). Mutations that alter hindbrain patterning such as *hoxa1*, impair FGF signaling from rhombomeres that lead to inner ear defects (Pasqualetti and Rijli, 2001). To determine if development of the rhombomeres is perturbed after *shox2* deletion, cell clusters corresponding to rhombomere progenitors were identified. Hierarchical clustering showed three major groups of progenitors from wild-type embryos. To classify progenitors from specific rhombomeres, we exploited the fact that rhombomeres can be identified by their *hox* gene regulatory networks. For each cluster, expression of the appropriate *hox* gene were identified. In *shox2^+/+^* embryos, rhombomere 5 (r5) was defined by *hoxb3a* and *hoxa4a* genes (Prince et al., 1998; Tambalo et al., 2020). Rhombomere 6 (r6) and 7 (r7) were defined by *hoxa2b* and *hoxd4a*, respectively. From hierarchical clustering, the dendrogram shows *shox2*^Δ*/*Δ^ cell populations corresponding to r5-7 that have altered *hox* gene expression. Wild-type embryos displayed individual clusters corresponding to rhombomere 5-7, while *shox2*^Δ*/*Δ^ embryos show three r6 clusters along with altered r5 and r7 clusters (Fig. 7D). Progenitors from rhombomeres of *shox2*^Δ*/*Δ^ also showed subtle changes in marker gene expression corresponding to ventral and dorsal/medial markers as previously described (Tambalo et al., 2020) (Fig. 7E). The scRNA-seq experiments showed transcriptome changes in otic progenitors and that rhombomeres are not developing normally after *shox2* ablation. These results suggests that *shox2* deletion can have cell autonomous or indirect effect on inner ear development.

**Fig. 8.**
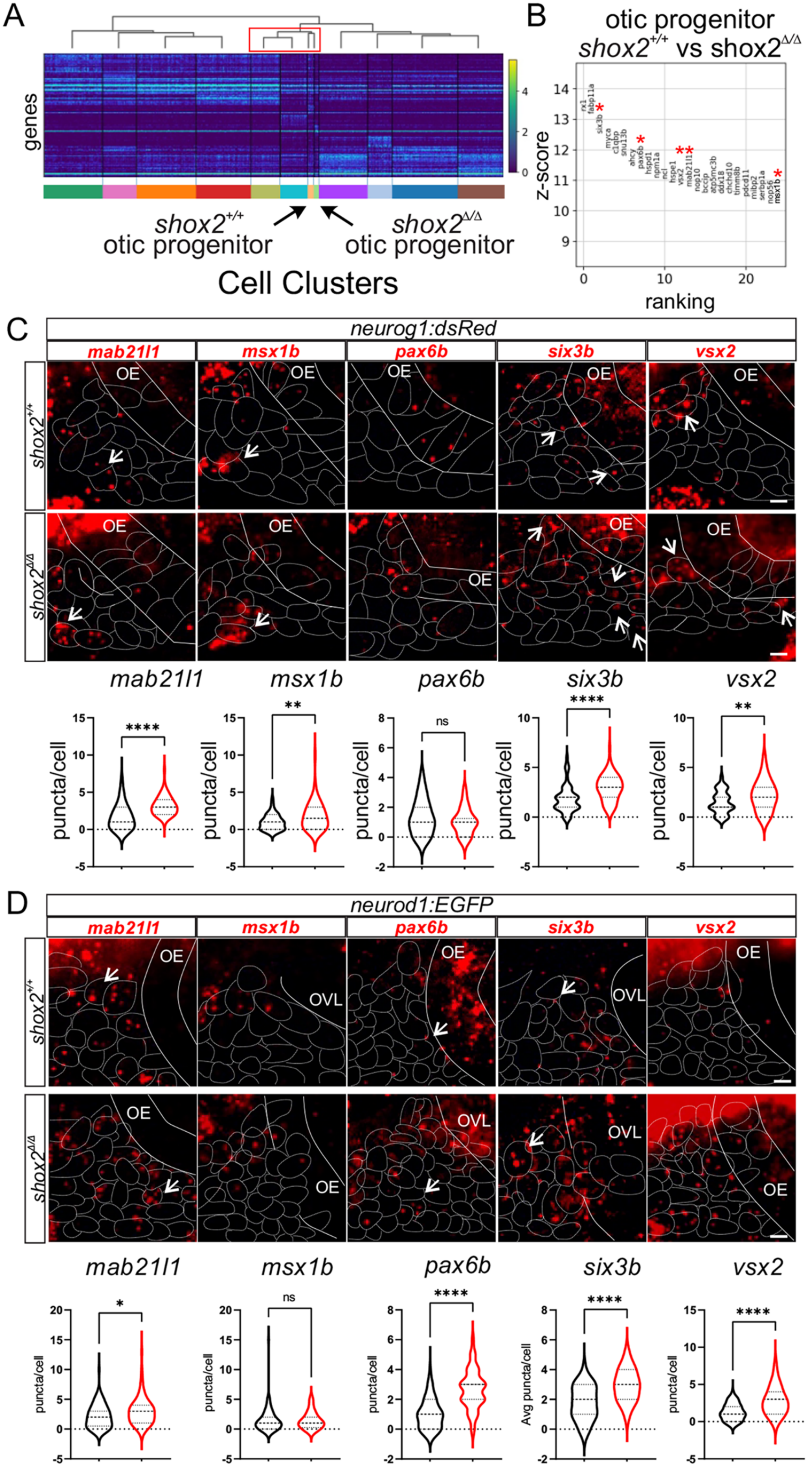
**Determining differential gene expression between *shox2^+/+^* and *shox2*^Δ*/*Δ^ otic progenitors and developing neurons.** (A) Heat map displaying transcript levels of the fifty variable genes for individual cells from each cluster identified in the progenitor population. Cells from each cluster is marked by a different color rectangle at the bottom of the heatmap. Arrows point to otic progenitor populations from shox2^+/+^ and shox2^Δ/Δ^ embryos. Dendrogram displays the similarity and relationship between cell populations. (B) Differential expression analysis of genes from *shox2^+/+^* otic progenitors with *shox2*^Δ*/*Δ^ mutant otic progenitor. The z-score associated with each gene represents the number of standard deviation away from the mean gene transcript levels between *shox2^+/+^* and *shox2*^Δ*/*Δ^ cells. The ranking of significantly altered genes is based on the z-score. Red asterisks denote transcripts that are subjected to smFISH. smFISH using *mab21l1*, *msx1b*, *pax6b*, *six3b* and *vsx2* probes was performed on 24 hpf *shox2^+/+^* and *shox2*^Δ*/*Δ^ embryos in (C) TgBAC(*neurog1:dsRed*) or (D) TgBAC(*neurod1:EGFP)* larvae. For TgBAC(*neurog1:dsRed*) lines, the following number of embryos were used for each smFISH probe: *mab21l1* (*shox2^+/+^*, *n*=15; *shox2*^Δ*/*Δ^, *n*=12), *msx1b*, (*shox2^+/+^*, *n*=15; *shox2*^Δ*/*Δ^, *n*=13), *pax6b* (*shox2^+/+^*, *n*=15; *shox2*^Δ*/*Δ^, *n*=13), *six3b* (*shox2^+/+^*, *n*=14; *shox2*^Δ*/*Δ^, *n*=13) and *vsx2* (*shox2^+/+^*, *n*=15; *shox2*^Δ*/*Δ^, *n*=12). For TgBAC(*neurod1:EGFP*) lines, the following number of embryos were used for each smFISH probe: *mab21l1* (*shox2^+/+^*, *n*=13; *shox2*^Δ*/*Δ^, *n*=12), *msx1b*, (*shox2^+/+^*, *n*=15; *shox2*^Δ*/*Δ^, *n*=12), *pax6b* (*shox2^+/+^*, *n*=15; *shox2*^Δ*/*Δ^, *n*=13), *six3b* (*shox2^+/+^*, *n*=15; *shox2*^Δ*/*Δ^, *n*=13) and *vsx2* (*shox2^+/+^*, *n*=15; *shox2*^Δ*/*Δ^, *n*=12). Quantification of puncta per cell from whole-mount zebrafish at 24 hpf in dsRed or EGFP marked cells displayed as violin plots from shox2^+/+^ (black) and shox2^Δ/Δ^ (red) embryos. Solid lines mark the otic epithelium (OE) and otic vesicle lumen (OVL) in the images, while dashed lines outline cells expressing the fluorescent transgenes. Values reported (mean±s.e.m.). Statistical tests were performed on the number or larvae from each sample using Welch's *t*-test. n.s. (not significant), *P≤0.05, **P≤0.01, ****P≤ 0.0001. Scale bars: 10 µm.

We wanted to validate some of the transcriptional changes in otic progenitors. Deletion of *shox2* results in three distinct mutant otic progenitors each with specific transcriptome changes. It is likely that alteration of an initial population of progenitors may result in additional changes and the appearance of new populations of aberrant otic progenitor cells. To determine the relationship between the otic cell types, hierarchical clustering was performed and a heatmap of the top 50 variable genes in each cell was used to display the relationship between each cell cluster. The associated dendrogram with the distribution of the clades in the dendrogram correspond to similarities between cell clusters (Fig. 8A). The otic progenitors were grouped together in the dendrogram. *shox2*^Δ*/*Δ^ cell cluster that displayed the highest similarity to the *shox2^+/+^* otic progenitors were used for differential gene analysis.

Differential gene expression analysis between the *shox2^+/+^* and *shox2*^Δ*/*Δ^ otic progenitor clusters from scRNA-seq data revealed altered gene expression. Genes that show the greatest differences between shox2^+/+^ and *shox2*^Δ*/*Δ^ otic progenitors were identified based on their z-score, a metric corresponding to the number of standard deviations away from the mean transcript levels. Using the z-score, a ranked list of differentially expressed genes was identified for pairwise comparison to wild-type and mutant otic progenitors, with genes implicated in specification and differentiation in other tissue types marked by red asterisks (Fig. 8B). To validate changes in transcript levels, we employed single molecule fluorescence *in situ* hybridization (smFISH) on 24 hpf embryos. We chose genes *mab21l1* (Wong and Chow, 2002), *msx1b* (Phillips et al., 2006), *pax6b* (Nornes et al., 1998), *six3b* (Seo et al., 1998) and *vsx2* (Vitorino et al., 2009) that were on the ranked list and have been implicated in cell fate specification or development in different tissues. To identify the cell population that could be affected in the inner ear, we used *Tg(neurog1:dsRed)* and *Tg(neurod1:EGFP)* embryos. smFISH probes were used to detect transcripts in *shox2^+/+^* and *shox2*^Δ*/*Δ^ embryos. Puncta corresponding to mRNA molecules in fluorescent cells were quantified with the genotype blinded to the experimenter. All transcripts showed altered expression levels in *shox2*^Δ*/*Δ^ embryos (*mab21l1*, *P*<0.0001; *msx1b*, *P*<0.01; *six3b*, *P*<0.0001; *vsx2*, *P*<0.01) except for *pax6b* (Fig. 8B). To mark developing neurons, the *Tg(neurod1:EGFP)* reporter was used. The same smFISH probes were used to detect transcripts in *shox2^+/+^* and *shox2*^Δ*/*Δ^. Quantification of puncta in marked developing neurons revealed changes in all transcripts in *shox2*^Δ*/*Δ^ embryos (*mab21l1*, *P*<0.05; *pax6b, P<0.0001; six3b*, *P*<0.0001; *vsx2*, *P*<0.0001) except of *msx1b* (Fig. 8C). These results validated the differential gene expression changes observed between *shox2^+/+^* and *shox2*^Δ*/*Δ^ otic progenitors from the scRNA-seq dataset. Some of the altered transcripts persist in developing neurons suggesting that altered transcript levels in mutant otic progenitor likely perturbs normal otic neuronal development.

## DISCUSSION

Within the inner ear, there are two main populations of neurons that transmit auditory and vestibular information. Genes involved in the development of either vestibular or auditory SAG neurons from otic progenitors is still unclear. Delamination along the anterior and posterior ends of the otic vesicle as well as differences in developmental timing likely allows integration of different patterning signals (Maier and Whitfield, 2014; Radosevic et al., 2011) to work in conjunction with intrinsic factors to specify for auditory or vestibular neuron fate. Here we show that *shox2*, a homeobox domain containing transcription factor, is required for establishing otic progenitor identity and subsequent development of anterior SAGs that correspond to vestibular neurons. Changes in cell identity was revealed through scRNA-seq. Multiple aberrant populations of otic and rhombomere progenitors from mutant fish likely contribute to abnormal vestibular neuron development. Interestingly, *shox2* deletion only affects the number of vestibular neurons and spares the auditory neurons. These changes may arise due to *shox2* function in pathways specific for vestibular neuron development.

We show that *shox2*^Δ*/*Δ^ larvae fail to inflate their swim bladders by 7 dpf. The absence of a functional swim bladder reduces survival of zebrafish larvae (Goolish and Okutake, 1999; Schoppik et al., 2017). The swim bladder in zebrafish allows the fish to counteract the density imposed by water and maintain buoyancy after changing depth in the water column (Heller and Brandli, 1999; Lindsey et al., 2010; Robertson et al., 2007; Schoppik et al., 2017). Furthermore, the swim bladder in developing zebrafish larvae is required for the maintenance of body orientation when swimming (Ehrlich and Schoppik, 2019; Schoppik et al., 2017). *shox2* expression has not been detected in the swim bladder, we propose that vestibular dysfunction occurs due to loss of anterior SAGs that innervate the utricle. The lack of a vestibular input could indirectly affect swim bladder inflation.

Larval zebrafish vestibular system is essential for controlling the nose-up and nose-down orientation, which allows larvae to swim to the surface, get air and inflate their swim bladders (Goolish and Okutake, 1999; Riley and Moorman, 2000; Schoppik et al., 2017). Behavioral data indicates that *shox2*-null larvae have difficulty maintaining their balance. During development, zebrafish larvae posture is unstable. To maintain postural control, they move in swim bouts (Bagnall and Schoppik, 2018; Ehrlich and Schoppik, 2019). We suggest that *shox2*-null larvae initially swim at greater distances than wild-type siblings to maintain postural control. The swim bout would allow the *shox2*-null mutant larvae to compensate for any balance deficiencies they might have as they develop. This behavior recovers to normal by 7 dpf and likely alludes to compensatory mechanisms for vestibular function as the fish matures.

The developmental role of zebrafish *shox2* in the inner ear has not previously been studied. Our data show that zebrafish *shox2* is expressed at early stages of SAG development starting in otic neuronal progenitors and persisting in developing neurons but disappears when anterior and posterior SAGs segregate. Ablation of *shox2* results in a significant decrease of anterior SAGs, a subset of SAGs that innervate utricular hair cells that convey vestibular information but leaves neurons that innervate the saccular hair cells unaffected. Cell lineage tracing has shown that there are three different neurosensory progenitor pools in the developing inner ear. Progenitors in the anterior region of the developing ear have segregated at the onset of *neurod1* and *atoh1* expression to establish neuronal and sensory precursors, respectively (Sapede et al., 2012). Expression of *shox2* overlaps with the establishment of neuronal precursors but only affects the number of vestibular neurons and do not display an obvious effect on auditory neurons. We acknowledge that a more subtle deficit may be present in auditory neurons but our results suggests that *shox2* is necessary for vestibular development soon after neuronal precursors are present in the anterior pole of the otic vesicle.

From the scRNA-seq data, we identified an otic neuronal progenitor population as previously reported (Tambalo et al., 2020). After ablation of shox2, three novel populations of otic progenitors in the mutant zebrafish arise that show altered expression of six1a, six1b and eya1. These populations likely correspond to abnormal otic populations. Multiple inner ear cell types may be affected but we observe that these changes ultimately decrease the number of anterior SAGs. These changes likely affect development of vestibular neurons as observed by decreased number of developing anterior SAGs in *shox2*^Δ*/*Δ^ embryos while leaving auditory neuron numbers unchanged. The *shox2* gene regulatory network may help precursors decide whether to become vestibular instead of auditory neurons. Similar to zebrafish, scRNA-seq of mouse developing otic cells from E9.5-15.5 also show that cochlear vestibular ganglion precursors express *Shox2*. Unlike zebrafish, *Shox2* is present in developing spiral ganglion neurons but not in vestibular neurons (Sun et al., 2022). The difference between mice and fish in determining vestibular and auditory neurons may be due to the lack of the murine *Shox* gene (Gianfrancesco et al., 2001). Zebrafish *shox* may provide a distinct gene regulatory network that contributes the differences observed in fish. Nevertheless, these studies place the role of *shox2* in development of inner ear neuron subtypes.

During embryogenesis, inner ear development is known to require external signals from the developing rhombomeres. Altered rhombomere development such as mutations in *hoxa1*, indirectly affect otic development (Pasqualetti et al., 2001). From single cell RNA-seq analysis, we find aberrant progenitor populations in developing rhombomeres 5-7 that suggest the rhombomeres are also not developing properly. Adjacent rhombomeres are known to provide instructional cues for otic vesicle patterning and development. Improper rhombomere patterning can ultimately alter signaling required for otic development and contribute to inner ear defects observed in *shox2*^Δ*/*Δ^. The lack of *shox2* could result from non-autonomous defects in otic development.

During otic development, the majority of early-born SAG precursors from the anterior region produce vestibular neurons, whereas posterior neurons that arise later include auditory neurons. The early expression of *shox2* at 18 somite stage (∼18 hpf) and its disappearance by 48 hpf during SAG development implicate a role for *shox2* regulating the decision for SAGs to become vestibular neuron. Establishing an anterior and posterior otic identity may be crucial in the decision to generate vestibular neurons. Fgf and Hedgehog (Hh) signaling pathways are well established and shown to specify anterior and poster identity in the otic placode and vesicle (Hammond and Whitfield, 2011). Inhibiting Hh signaling results in loss of posterior otic structures and are replaced by duplicated anterior otic structures. Conversely, increased activation of Hh signaling by overexpressing sonic hedgehog (shh) in the embryo results in absent anterior otic structures and duplicated posterior structures (Hammond et al., 2003). Hh genes are expressed between 16.5-30 hpf in midline tissues, notochord and floorplate, these include shh in both the notochord and floorplate, tiggy-winkle hedgehog (twhh) in the floorplate and echidna hedgehog (ehh) in the notochord (Hammond et al., 2003). This time point coincides with when we observe *shox2* reporter expression around the developing otic placode at 18 somite state (∼18 hpf) and around the otic vesicle at 24 hpf. The shox2 reporter signal disappears by 48 hpf. Deletion of *shox2* may affect a gene regulatory network in otic cell types that activates Hh signaling resulting in a reduction of anterior SAGs.

Fgf has been implicated in initial neuroblast specification in multiple vertebrate species and likely plays a role in parallel with *shox2* to establish the appropriate number of vestibular neurons. A gradient of Fgf levels coordinates distinct steps in SAG development which includes specifying neuroblasts within the otic vesicle. *shox2* likely acts within this timepoint as neuroblasts are being specified to help determine vestibular neuron identity. Rising levels of Fgf terminate further specification (Vemaraju et al., 2012). Alteration in expression of these genes was observed from differential gene expression from scRNA-seq data show genes associated with FGF signaling. In mammals, a gene regulatory network controlled by FGF signaling during inner ear development shows that FGF affects *Pax6* and *Six3* (Anwar et al., 2017). In left right asymmetry, FGF signaling upstream of transcription factors such as *six3b* and ectopic activation of FGF signaling leads to overexpression of *six3b* while reduction in FGF signaling leads to decreased *six3b* expression (Neugebauer and Yost, 2014). We show by smFISH that *six3b* is upregulated and suggest that FGF signaling may be deregulated in *shox2* mutant fish that leads to improper development of anterior SAGs resulting in the reduction of vestibular neurons. The aberrant populations of cells observed in the scRNA-seq could be the result from acquiring abnormal differentiation trajectories or correspond to populations of neurons that are stalled at early developmental steps. Overall, the data suggests that *shox2* may act downstream or in parallel with FGF and Hedgehog signaling for vestibular neuron development.

Here, we implicate a role of *shox2* in zebrafish vestibular neuron development. *In situ* hybridization shows the presence of shox2 expressing cells that reside in the otic placode that corresponds to neurosensory progenitors. Lineage mapping of a *shox2* reporter marked neuronal and sensory progenitors in the developing inner ear. Molecular analysis confirms the presence of aberrant otic progenitors that ultimately results in a reduction of anterior SAGs. Together, our findings implicate *shox2* in vestibular neuron development.

## MATERIALS AND METHODS

### Zebrafish husbandry and strains

Adult *Danio rerio* (zebrafish) were housed under standard conditions at the Rutgers University aquatic facility in Nelson Biology Laboratories. Zebrafish embryos were obtained by natural spawning of sexually mature adults. Embryos were collected and kept in 60 µg/ml of Instant Ocean Sea Salt solution supplemented with Methylene Blue at 28.5°C (Westerfield, 2000). Embryos used for fluorescence microscopy were incubated in 60 µg/ml Instant Sea Salt solution without Methylene Blue or in E3 medium (5 mM NaCl, 0.17 mM KCl, 0.33 mM MgSO_4_, 0.33 mM CaCl_2_ pH 7.2 buffered with HEPES) and supplemented with 0.176 mM propilthioluracil (PTU) (Kindt et al., 2012; Westerfield, 2000). Reporter lines used were kept as separate inbred strains.

To label all inner ear cell types, *Tg(pax2a:GFP)^e1Tg^* was used (Picker et al., 2002). Inner ear neuronal progenitors and transit-amplifying neuronal population were identified using *TgBAC(neurog1:dsRed)^nl6Tg^* and *TgBAC(neurod1: EGFP)^nl1Tg^* reporter zebrafish, respectively (Drerup and Nechiporuk, 2013; Obholzer et al., 2008). Fluorescent reporters containing the *shox2*^Δ^ allele were kept in a mixed genetic background. The following *reporters* were generated containing the *shox2*^Δ^ allele: Tg*(shox2*^Δ*/+*^*, pax2a:GFP)^e1,^, Tg(shox2*^Δ*/+*^*, neurod1:EGFP)*^nl1^, *Tg(shox2*^Δ*/+*^*, neurog1:dsRed)^nl6^* and Tg*(shox2*^Δ*/Gal4*^*,UAS:GFP)^nns51Tg^*. Experiments performed with wild-type embryos were done with EKW and AB strains. Embryos were staged accordingly (Kimmel et al., 1995). All experiments were approved by the Institutional Animal Care and Ethics Committee of Rutgers University. Table 2 for genetic background information and resource identification number.

**Table 2.**
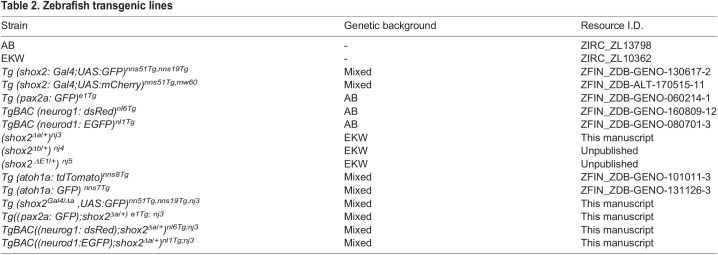
Zebrafish transgenic lines

### Generating shox2 mutant and shox2 reporter animals

*shox2* mutant allele was generated using single guide RNA complexed with CRISPR/Cas9. sgRNAs targeting exons 1 and 2 of the *shox2* gene were designed using CRISPRscan (Moreno-Mateos et al., 2015). A combination of sgRNAs (Table 1) was used to target exons 1 and 2 of the *shox2* gene. CRISPR/Cas9 sgRNA complexes were formed by incubating a NLS-Cas9 protein and the pair of sgRNAs at room temperature for 10 min. Approximately 483 pg of Cas9 with 75 ng of both sgRNA was microinjected into EKW zebrafish embryos at 1-4 cell stage. Founder fish with *shox2*^Δ^ allele were identified by PCR genotyping and outcrossed to wild-type EKW adults. *shox2*^Δ^ PCR product was cloned into pCRII and sequenced. F1 zebrafish with the predicted deletion were propagated. Three different *shox2* mutant alleles were identified and characterized. F1 zebrafish that harbored the *shox2*^Δ^ allele was used to generate F2 animals. The F2 fish were maintained as *shox2*^Δ*/+*^ heterozygotes.

To generate the shox2:Gal4 knock-in transgenic zebrafish, a CRISP/Cas9-mediated genome engineering method was employed (Kimura et al., 2014). A donor plasmid containing a short guide RNA (sgRNA) (5′-GGGGCTCGCGGTGAGGGAAGG-3′) was used to target a region upstream of the *shox2* protein coding sequence. The donor plasmid also harbored a minimal heat-shock protein 70 (hsp70) promoter sequence with the *Gal4* gene. The donor plasmid was co-injected with Cas9 mRNA. Cleavage by CRISPR/Cas9 and homology independent DNA repair inserted the minimal promoter and Gal4 gene into the region. To generate reporters, Tg(shox2:Gal4) fish were mated with transgenic fish carrying UAS:GFP or UAS:mCherry. These animals, *Tg(shox2:Gal4; UAS:GFP)^nn51Tg^ and Tg(shox2:Gal4; UAS:mCherry)^nn51Tg;mw60^* reporters were used for labeling *shox2* cells as described (Marquart et al., 2015; Miesfeld and Link, 2014).

### PCR genotyping of shox2 Alleles

Genomic DNA was obtained from either adult zebrafish fins, fixed tissue, or fresh embryonic tissue. The tissue was lysed using 1X DNA extraction buffer (10 mM Tris HCl pH 8.3 and 50 mM KCl) and freshly added 0.3% IGEPAL, 0.3% Tween-20) (JIng, 2012). Tissue was incubated in 1X DNA extraction buffer at 95°C to lyse cells and incubated in 1X DNA extraction buffer containing 1 µg/µl of proteinase K was added at 55°C for 16 h. To inactivate the proteinase K, samples were incubated at 95°C for 10 min before use. Extracted genomic DNA was used for PCR genotyping. To decrease secondary structure in the genomic DNA, samples were heated to 95°C for 5 min and immediately placed on ice before using for PCR. The primers were incubated at 70°C for 5 min and then placed on ice. PCR reactions were set up by using EconoTaq Plus Green 2X Master Mixes (Lucigen), genomic DNA (0.5 μl) and primer (100 nM of each primer) as suggested by the manufacturer. PCR reactions were incubated at 95°C for 20 s, 60°C for 20 s and 68°C for 30 s for 40 cycles. The *shox2^+^* allele was identified by PCR amplification using *shox2WT F* and *shox2WT R* primers, while the *shox2*Δ allele was detected using *shox2*Δ*F* and *shox2*Δ*R* primers that flank the deletion site. Sequence of primers and amplicon sizes are listed in Table 1.

### mRNA expression analysis

Embryos were manually dechorionated or treated with 1 mg/ml Pronase E before rinsing three times with embryo water. Embryos were anesthetized with 0.03 mM of MS-222 in the Instant Ocean Salt solution. Embryos were deyolked by pipetting the embryos up and down in Ringers Buffer (116 mM NaCl; 2.9 mM KCl; 5.0 mM HEPES at pH 7.2) and 1 mM EDTA and rinsed twice in cold Ringers Buffer. A piece of the tail was clipped from each embryo for PCR genotyping before using the remaining embryo for RNA extraction. Individual embryos were placed in 1.5 µl microcentrifuge tubes and incubated at −80°C for at least 30 min for long term storage and cell lysis before RNA extraction.

The total RNA was obtained by crushing the frozen embryos in disposable pre-chilled pestles. A total of 300 µl of TRIzol (Thermo Fisher Scientific) was used per embryo (De Jong et al., 2010). 60 µl of chloroform per 300 µl of TRIzol was added to the samples to separate the organic and aqueous phases. 150 µl of isopropanol per 300 µl of TRIzol was added to each sample to precipitate the RNA. The isopropanol was supplemented with 15 µg/ml of GlycoBlue (Thermo Fisher Scientific) to aid with RNA precipitation. Total RNA was quantified using a NanoDrop spectrophotometer. cDNA was synthesized by using 0.5ug of total RNA together with the qScript cDNA synthesis kit (Quanta Bio) according to the manufacturer's instructions. cDNA synthesis reaction was incubated with RNaseH for 30 min at 37^°^C.

Relative levels of cDNA were measured by quantitative real-time PCR (qPCR) using SYBR green (Life Technologies). qPCR mix containing cDNA (1 µl), primers (300 nM of each primer), and SYBR green master mix was incubated in the StepOnePlus or Quant Studio3 (Thermo Fisher Scientific) real-time PCR machine at 95°C for 15 s, 60°C for 1 min for 40 cycles. Each biological replicate contained technical triplicates. Samples were normalized to the *eef1a1l1* and compared to controls. Relative differences in cDNA levels were calculated using the ΔΔCT method. Primers used for qPCR are listed in Table 1.

### *In situ* hybridization of zebrafish embryos

The *shox2* anti-sense probe for *in situ* hybridization was obtained by *in vitro* transcription of the full length *shox2* in the CMV SPORT6.1 plasmid backbone. The plasmid was linearized using KpnI, purified with a DNA concentrator −5 column (Zymo Research), and used as DNA template for *in vitro* transcription. 1 µg of purified plasmid was incubated with 30 units of T7 RNA polymerase containing either a fluorescein or a digoxigenin (DIG) RNA labeling mix (Perkin Elmer). The reaction was incubated at 37°C for 3 h and stopped by the addition of EDTA. RNA probes were purified from free nucleotides using NucAway spin columns (Thermo Fisher Scientific) before use as probe for *in situ* hybridization.

Embryos for *in situ* hybridization were fixed in fresh 4% formaldehyde in 1X PBS (137 mM NaCl, 2.7 mM KCl, 10.1 mM Na_2_HPO_4_, 1.8 mM KH_2_PO_4_; pH 7.4). After fixation, embryos were washed for 10 min in 1X PBS/0.1% Tween-20. Samples were incubated in an increasing methanol series at room temperature with 25%, 50%, 75% methanol, for 10 min at each step. Methanol solutions were diluted in 1X PBS/0.1%Tween-20. Samples were finally dehydrated for 10 min in 100% methanol and stored at −20°C overnight. Samples were re-hydrated in 75%, 50%, and 25% methanol diluted in 1X PBS/0.1%Tween-20. Re-hydrated samples were washed in 1X PBS/0.1%Tween-20 and permeabilized with 1X PBS/0.1%Tween-20 containing 10 µg/ml Proteinase K. Incubation time in Proteinase K solution depended on the age of the zebrafish embryos (Westerfield, 2000). To remove Proteinase K, samples were washed twice for 5 min in 1X PBS/0.1%Tween-20, post-fixed for 30 min at room temperature with 4% formaldehyde, washed twice for 5 min each time in 1X PBS/0.1%Tween-20 and incubated in pre-hybridization buffer (50% formamide, 5× SSC, 50 μg/ml heparin sodium salt, 0.1% Tween-20, 5 mg/ml torula RNA) for 2-3 h from 68-70°C. Labeled probes were diluted into pre-hybridization buffer, added to samples and incubated overnight at 68-70°C. Samples were subjected to a series of washes in Saline Sodium Citrate (SSC) based solutions (1X SSC: 150 mM NaCl; 15 mM Na_3_C_6_H_5_O_7_, pH 7.0) at 68-70°C. Samples were washed twice for 30 min in 50% formamide/ 2X SSC, once for 15 min in 2X SSC and once for 30 min in 0.2X SSC. Samples were washed twice for 5 min at room temperature in 1X maleic acid buffer/0.1%Tween-20 (1X MAB: 50 mM C_4_H_4_O_4_, 75 mM NaCl) before incubating in blocking buffer (2% Blocking reagent, 10%normal goat serum (NGS), 1X MAB, 0.1%Tween-20) for 1 h at room temperature. Anti-DIG-AP antibody was added to the blocking buffer and samples were incubated overnight at 4°C. To develop the samples, specimens were washed four times for 20 min each time in 1X MAB/0.1% Tween-20, washed twice for 5 min in 1X PBS and incubated at 4°C in the shaker with NBT/BCIP. Excess chromagen was removed in two washes of 10 min in 1X PBS and incubated overnight in 50%Glycelol/ 1X PBS (Thisse et al., 1993). Samples were then mounted on 1% low melting point agarose and imaged with an Olympus SZX16 stereo microscope. For frozen sections, embryos at 18 somite stage were embedded in OCT and frozen. Frozen blocks containing embryos were cut into 20 µm sections on a cryostat at −20°C mounted on a glass coverslip and images acquired on an Olympus BX63 histology microscope.

### Whole-mount immunostaining of zebrafish embryos

Zebrafish embryos were fixed at the desired stages in 4% formaldehyde for 4 h at 4°C (Kindt et al., 2012). Then embryos were postfixed for 1-3 h at room temperature in fresh 4%formaldehyde. Alternatively, embryos were fixed overnight at 4°C. To remove excess formaldehyde embryos were washed five times in 1X PBS/0.01% Tween-20 for 5 min each. The immunohistochemistry of zebrafish embryos was modified from a published protocols (Andermann et al., 2002). Briefly, samples were washed three times for 5 min in 1X PBS/ 0.1%Triton X-100. Subsequently, embryos were washed three times for 30 min in 10 mM Tris-Base pH 7.4. Samples were blocked for 1-2 h at room temperature in blocking buffer (1X PBS, 2%NGS, 1% bovine serum albumin (BSA), 1% DMSO, and 0.1% Triton- X100). Primary antibodies were incubated overnight at room temperature. After antibody incubation samples were washed three times for 15 min in 1X PBS/0.1%Triton-X100. Secondary antibodies and Hoechst (500 ng/μl) were diluted in the blocking buffer and incubated overnight at room temperature. After washing excess secondary antibodies, samples were incubated overnight at 4°C in 50%Glycerol/1X PBS. Samples were mounted on glass bottom dishes using 1% low melting agarose to immobilize zebrafish embryo samples. 50%Gycerol/ 1X PBS was added to the dish for imaging. Antibodies are listed in Table 3.

**Table 3.**
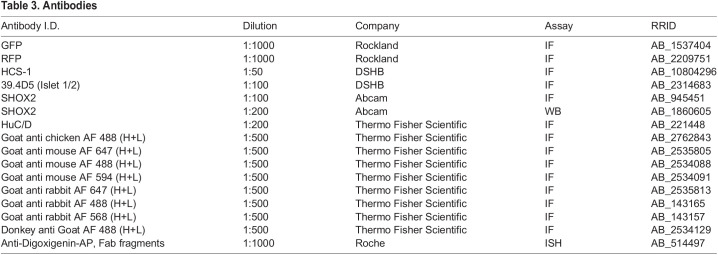
Antibodies

### Quantifying zebrafish hair cells and SAG

Images for quantification of SAG in the zebrafish inner ear were acquired with the Zeiss LSM 800 confocal microscope with a 20X air objective. A digital zoom of 1.5X −5.0X was used. Digital zoom varied depending on the developmental stage and region imaged. Z-stacks were acquired through the entire inner ear at all developmental stages in 1.5-3.0 µm steps. ImageJ was used for quantification of fluorescence images. GFP labeling from the *neurod1* reporter was used to identify the aSAG and pSAG around the otic vesicle. SAG quantification was performed by identifying the optical section containing the brightest Shox2 labeling within a Z stack. Fluorescence from the immunolabeling was accomplished by drawing a region of interest (ROI) around individual HuC/D positive cells in the developing SAG.

### Swim bladder inflation and balance assay

These assays were all performed blinded to genotype. At 4 dpf individual zebrafish larvae were placed on a 24-well plate (1 larva/well). Swim bladder inflation was scored from 4-7 dpf. If the swim bladder was not fully inflated, it was counted as a non-inflated swim bladder. To determine behavioral abnormalities related to balance, only zebrafish with fully inflated swim bladders were used. The zebrafish larvae were placed on a 60 mm petri dish filled with approximately ∼15 ml of embryo water and prodded on the lateral side of their trunk with an eyelash tool. The distance moved, and the larvae's body position relative to the bottom of the dish were recorded. To determine if the larvae can maintain balance, the fish were observed for 1 min. They were scored as ‘pass’ if the larvae maintained a ventral-up position relative to the bottom of the dish. The fish would be scored as ‘fail’ if, they could not maintain a ventral position within the trial period (Kwak et al., 2006). After the behavior assay was completed, larvae were genotyped.

### Acoustic startle response

The acoustic startle response was later elicited in a chamber modified from a 96–well plate with a speaker attached to the bottom. To test for an acoustic startle response in zebrafish, individual 5 dpf larvae were placed in the central 24 wells of a 96-well plate (1 larvae/well) and housed in a sound-attenuating chamber. A Visatron SC 5.9 ND speaker was placed 4 cm away from the multi-well plate. The speaker was connected to the RZ6 Multi-I/O Processor to produce acoustic stimuli. Tones at 90 dB were presented as 24 ms cosine-squared gated 100 ms tone pips at 0.1, 0.2, 0.3, 0.5 and 1 kHz (Bhandiwad et al., 2013; Zeddies and Fay, 2005). A camera was placed over the multi-well plate to record larvae movement during tone presentation at 30 frames s^–1^ (1280×720 pixels resolution). A custom macro was written. The image analysis algorithm is based upon subtraction of images acquired before and after the presentation of a tone-burst to identify movement (Lin et al., 2016). Animals that showed movement 100 ms after tone burst were scored as showing a startle response. After the trials, zebrafish were sacrificed with a lethal dose of MS-222 and placed in lysis buffer for PCR genotyping.

### scRNA-seq of zebrafish otic region

To generate the embryos for scRNA-seq several genetic crosses were implemented. *shox2^+/+^* animals with neuronal progenitors and neuroblast labeling *Tg(neurog1:dsRed)* and *Tg(neurod1:EGFP)* animals were crossed to each other. Only embryos with both dsRed and EGFP labeling were retained for the experiment. *Tg(shox2^Gal4/^*^Δ*a*^*; UAS:GFP)* in-crosses produced GFP negative *shox2*^Δ*/*Δ^ and GFP positive *Tg(shox2^Gal4/a^; UAS:GFP)* embryos. GFP negative *shox2*^Δ*/*Δ^ embryos were used. Embryos were obtained at 16, 20 and 22 hpf. For each genotype, 40-60 sorted embryos were obtained. The difference in time points allowed for collection of different cells undergoing development within this time window.

Embryos were dechorionated with 1 mg/ml pronase E and 1X HBSS and rinsed two twice with de-yolking solution (0.03 mM MS-222 and 1X DMEM, high glucose and 1X B27), The embryos yolk was mechanically dissociated by gently pipetting the embryos in the de-yolking solution. Removing the yolk allows lateral mounting of the embryo. Embryos were rinsed three times in de-yolking solution, placed on its side to clearly identify the otic region was identified using oblique illumination under an Olympus SZX16 dissecting stereomicroscope equipped with a 0.8X objective with up to 11.5X magnification. The embryo was placed onto a Petri dish with a droplet of solution and excess liquid was wicked away from the embryo to maintain the position of the embryo. The embryo was then immobilized using a 0.33 mm fine tip needle. Lateral incisions flanking the otic vesicle were sequentially made using another 0.33 mm fine tip needle before a final cut released the tissue chunk from the embryo (Tambalo et al., 2020). Debris surrounding the tissue fragment was removed without damaging the otic vesicle and the tissue inspected for its integrity. Tissue fragments containing the intact otic region was transferred with a fire-polished glass pipette in a 0.5 ml Lo-bind tube coated with 10%BSA in 1X PBS (Wagner et al., 2018). Dissected tissue was kept on ice in de-yolking solution until cell dissociation. Tissue was washed with 1X HBSS, incubated at room temperature with 200 µl of FACSmax (Gelantis) supplemented with 1 mg/ml of activated papain (Worthington) for 5 min and mechanically dissociated with 10%BSA/1XPBS coated tips (Wagner et al., 2018). The dissociated cells were centrifuged at 300 g for 5 min at 4°C. The dissociation solution was removed, and cells were washed twice with 1% BSA/1XPBS. Cells were counted using the Moxi Z automated cell counter (Orflo) using the Type S cassettes before the final spin. Cells were resuspended at 1200 cells/µl in 0.05%BSA/ 1X PBS and placed on ice.

For each Single-cell cDNA libraries were generated using the Chromium Next GEM Single Cell 3′ GEM, Library & Gel Bead Kit v3 (10X Genomics). Approximately 20,000 dissociated cells were loaded onto a cassette in the Chromium Controller with accompanying reagents to generate GEMs. GEMs were subjected to reverse transcription to generate single-cell cDNA libraries before the oil emulsion was disrupted and cDNA purified using Dynabeads MyOne Silane (Thermo Fisher Scientific). cDNA was amplified by PCR for 11 cycles and purified using SPRIselect reagent (Beckman Coulter). cDNA fragmentation, A-tailing, and end repair was performed followed by adapter ligation of unique Illumina Index 1 (i7) adapters and Index 2 (i5) adapters for multiplexed paired-end sequencing. An additional 13 cycles of PCR were performed to amplify the libraries before samples were purified using SPRIselect reagent and used for sequencing.

Raw sequencing data were converted count matrixes using the 10X Genomics Cell Ranger software. The bcl files were demultiplexed into read1, read2, and index fastq files for each sample using the cellranger mkfastq command. Read counts from the set of fastq files were provided as input using the cellranger count command, which partitioned reads into the originating cell based on the 16 bp cell barcode. Reads were aligned to a custom reference transcriptome based on the zebrafish reference transcriptome (ENSEMBL GRCz11, release 98) and quantified for an annotated gene for each cell. The 10-base pair unique molecular identifier (UMI) was used to collapse PCR duplicates and accurately quantify the number of transcripts captured for each gene in individual cells. Both cellranger mkfastq and cellranger count were run with default options with an expected cell number of 10,000. The output resulted in an expression matrix (genes x cells) of UMI counts for each sample. Secondary analysis was performed using Scanpy. Cells were selected according to the following criteria. Cells containing less than 500 genes were removed. Genes have to be at least expressed in a minimum of three cells using the following commands: sc.pp.filter_cells(adata, min_genes=500), sc.pp.filter_genes(adata, min_cells=3). Next cells containing <6500 genes and <5% of mitochondrial-encoded genes were filtered using the following commands: adata=adata[adata.obs.n_genes_by_counts<6500, :], adata=adata[adata.obs.pct_counts_mt<5, :]. Cells containing a high percentage of mitochondrial genes are indicators of cellular stress and apoptosis. Among the remaining cells, the median number of UMIs per cell was ∼2000, and the median number of genes was 1000. These cells were used to select for highly variable genes using a dispersion-based method using the following command: sc.pp.highly_variable_genes(adata, min_mean=0.0125, max_mean=3, min_disp=0.5). The residual matrix was then scaled, centered, and used for the selection of variable genes for principal component analysis (PCA). After dimensionality reduction, Leiden graph-based clustering and different visualization were performed to identify cell populations and differentially expressed genes (Wolf et al., 2018).

### Whole-mount RNAScope and immunohistochemistry

Zebrafish embryos were collected at 24 hpf and fixed overnight at 4°C with fresh 4% formaldehyde. Samples were then washed with 1x PBS/ 0.01% Tween-20 and dehydrated in 25%, 50%, 75% methanol solutions diluted in PBS/0.1%Tween-20. Samples were finally placed in 100% methanol and incubated overnight at −20°C. Samples were brought to room temperature and air dried before performing RNAScope assays (Gross-Thebing, 2020). Embryos were then re-hydrated in 75%, 50%, and 25% methanol diluted in 1X PBS/0.1%Tween-20. Re-hydrated samples were washed in 1X PBS/0.1%Tween-20. Samples were then treated with the RNAScope hydrogen peroxide solution for 10 min and then rinsed in 1X PBS/ 0.01% Tween-20. Embryos were permeabilized by incubating in the RNAScope protease III for 20 min at room temperature. Embryos were washed in 1X PBS/ 0.01% Tween-20 and post fixed in 4% formaldehyde at room temperature. RNAScope probes were diluted to the manufacturer's instructions and incubated with embryos overnight at 40°C. Samples were washed with 0.2X SSC/0.01% Tween 20 before subjected to RNAScope amplification and signal development with the following modifications: 1X RNAScope wash buffer was replaced with 0.2x SSC/0.01% Tween 20. RNAScope assay was followed by immunohistochemistry against EGFP or dsRed. Before immunohistochemistry samples were washed twice for 10 min at room temperature with 1X PBS/ 0.01% Tween-20. Immunohistochemistry was performed as described above. To acquire images, zebrafish embryos were mounted and acquired with the LSM 800 confocal microscope with a 20X air objective using a 2X digital zoom. Quantification of acquired images was done with ImageJ. A maximal z projection 3-5 optical sections were used to identify EGFP and dsRed marked cells, as well as the number of puncta per cell. Cell boundaries were determined by cytoplasmic EGFP/ dsRed labeling. RNAScope puncta was manually counted blinded to genotype of the embryos.

## Supplementary Material

10.1242/biolopen.059599_sup1 Supplementary information
