# Supplementary material for: shox2 is required for vestibular statoacoustic neuron development
Source: Biol Open. 2023 Jan 3;11(12):bio059599. doi: 10.1242/bio.059599 (PMC9838637; doi:10.1242/bio.059599)
Supplement: Supplementary information [file biolopen-11-059599-s1.pdf]

Fig. S1

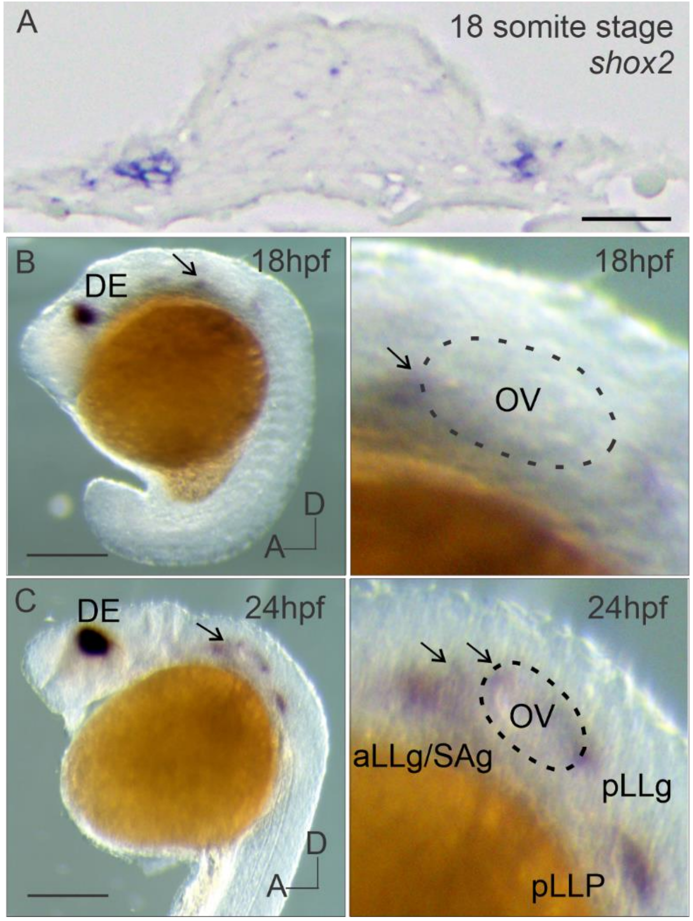

Fig. S1. ***In situ*** **hybridization of** ***shox2*** **transcript in the developing zebrafish inner ear** **(A)** *shox2 in situ* hybridization signal in a section of 18 somite stage (~16 hpf) embryo across the otic placode. Lateral view of whole mount *shox2 in situ* hybridization signal in zebrafish embryos (left panels) with magnified images of the otic vesicle region (right panels) at **(B)** 18 and **(C)** 24 hpf. Arrows point to the anterior ventral region of the otic vesicle. The dashed line in magnified images marks the otic vesicle (18,24 hpf). OV (otic vesicle), DE (diencephalon), aLLG (anterior lateral line ganglia), SAG (statoacoustic ganglion) pLLG (posterior lateral line ganglia) and posterior lateral line primordium (pLLP) are depicted. Anterior (A) and dorsal (D) axis for embryos are labeled. Scale bar: 50 µm.

Biology Open • Supplementary information

Fig. S2

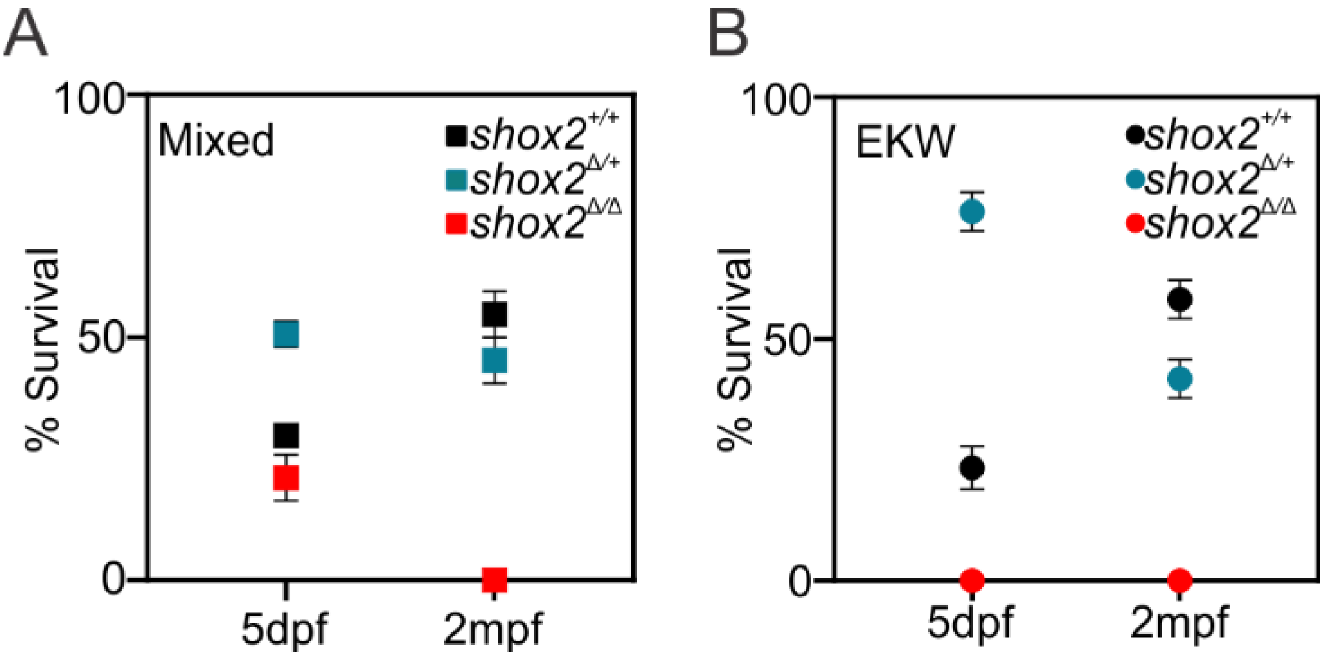

Fig. S2. Percent survival of *shox2* zebrafish cohorts

Cohort of fish from heterozygote incrosses in different genetic backgrounds. Percentage of surviving *shox2*$^{+/+}$ (black), *shox2*$^{\Delta/+}$ (green) and *shox2*$^{\Delta/\Delta}$ (red) at 5 dpf and 2 months of age in a (A) mixed genetic background and (B) in the EKW background.

Biology Open • Supplementary information

Fig. S3

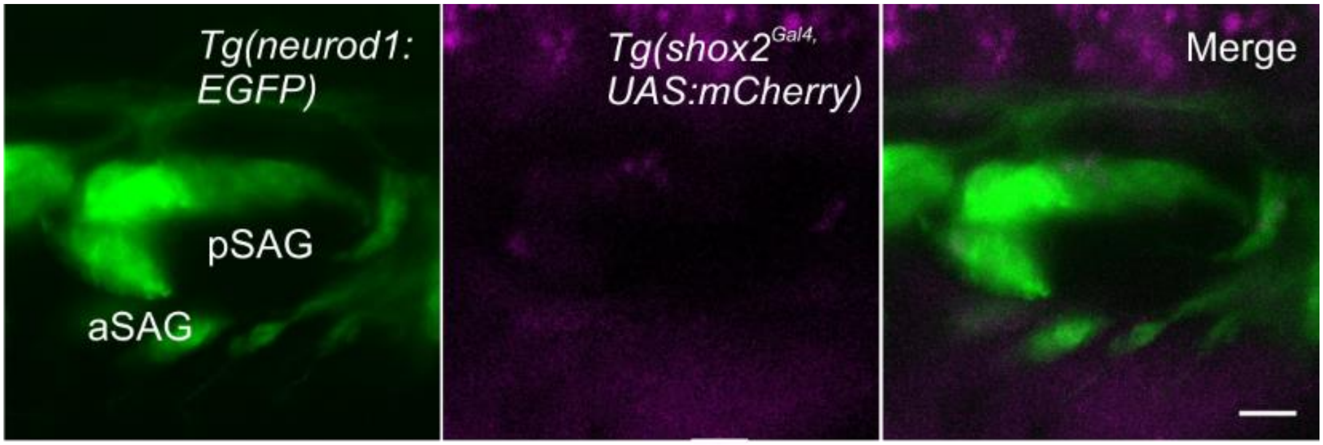

Fig. S3. Lineage labeling in developing statoacoustic ganglion at 48hpf using *shox2* fluorescent reporters.

Fluorescent images of developing inner ear from larvae containing *Tg (shox2^{Gal4}, UAS:mCherry)* (magenta) and *Tg(neurod1: EGFP)* (green) neuronal reporter at 48hpf. Merged image shows no *shox2* reporter labeled cells in either anterior or posterior statoacoustic ganglion neurons (n= 5).

Biology Open • Supplementary information

Fig. S4

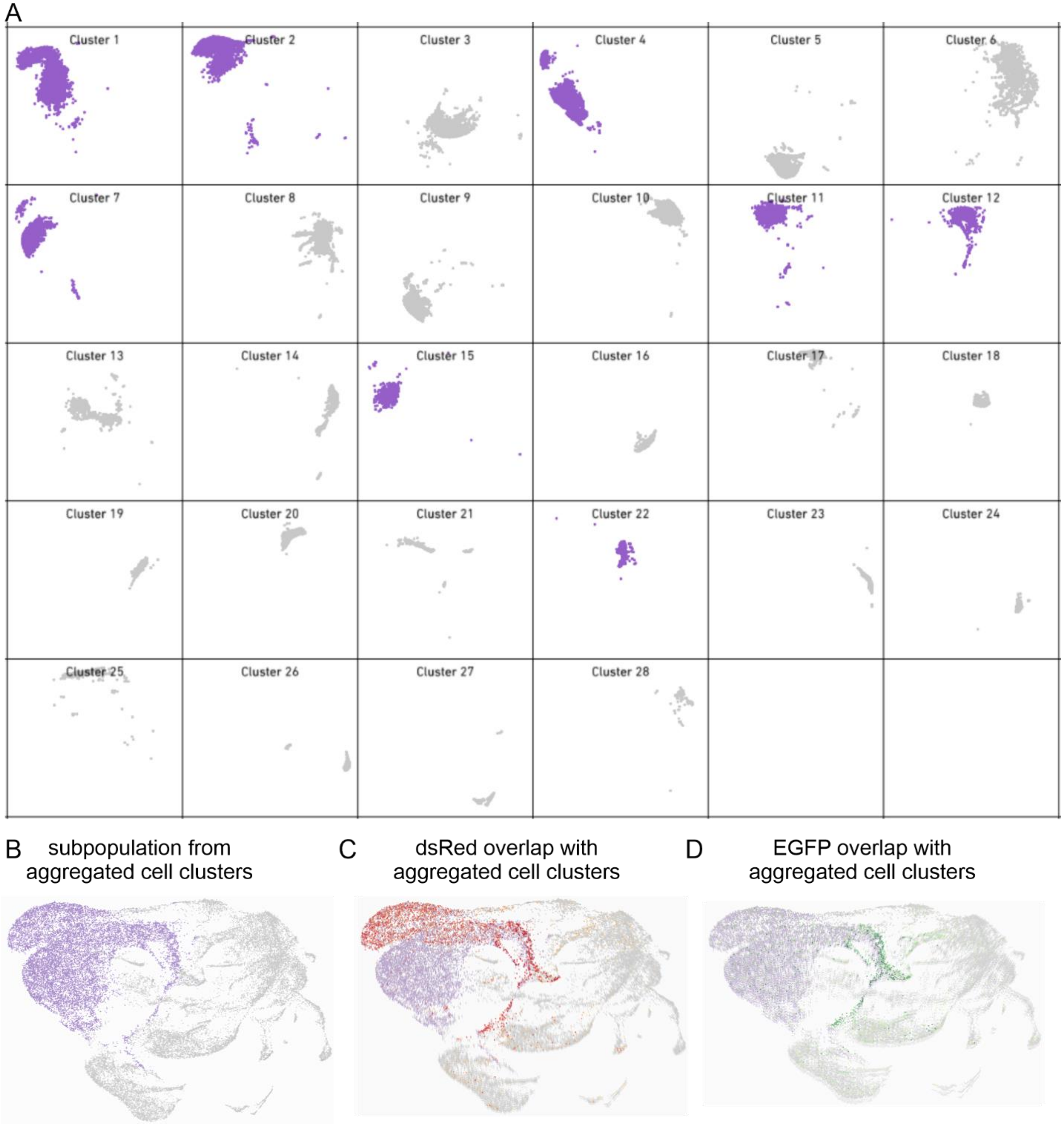

Fig. S4. Identification of progenitors and developing neurons

Cell clusters from the aggregate UMAP plot that containing both *shox2*$^{+/+}$ and *shox2*$^{\Delta/\Delta}$ cells were selected based on their overlap with cell clusters that express dsRed and EGFP. **(A)** Individual cell clusters from aggregate plot were split and individually shown. Cell clusters that overlapped with with dsRed and EGFP expres cells were identified and highlighted. **(B)** The aggregated plot obtained from individual cell clusters was overlaid with cells expressing **(C)** dsRed or **(D)** EGFP to confirm that the appropriate cell clusters were selected.

Biology Open • Supplementary information
